# Observation of erratic non-Hermitian skin localization and transport

**DOI:** 10.1038/s41467-026-76908-3

**Published:** 2026-08-21

**Authors:** Jia-Xin Zhong, Jee Woo Kim, Stefano Longhi, Yun Jing

**Affiliations:** 1 Key Laboratory of Modern Acoustics and Institute of Acoustics, Nanjing University, Nanjing, China; 2 Graduate Program in Acoustics, The Pennsylvania State University, University Park, Pennsylvania, PA USA; 3 Dipartimento di Fisica, Politecnico di Milano, Piazza Leonardo da Vinci 32, Milano, Italy; 4 IFISC (UIB-CSIC), Instituto de Física Interdisciplinar y Sistemas Complejos, Palma de Mallorca, Spain

**Keywords:** Topological matter, Statistical physics

## Abstract

Localization is a pervasive phenomenon across physics, shaping transport from electrons in solids to light and sound in engineered media. In traditional settings, disorder strongly impedes transport, resulting in dynamical localization or, at best, sub-ballistic or diffusive dynamics. A distinct regime, erratic non-Hermitian skin localization (ENHSL), can arise in globally reciprocal non-Hermitian lattices with disorder. It features macroscopic, disorder-dependent localization at irregular bulk positions with subexponential decay, linked to stochastic interfaces governed by the universal order statistics of random walks. We realize this regime experimentally in an acoustic lattice implementing a disordered Hatano-Nelson chain with imaginary gauge fields. Using Green’s-function-based spectroscopy together with time-resolved measurements on the same platform, we reconstruct the full complex spectrum and eigenstates, and directly observe wave-packet dynamics. We observe ballistic transport despite strong spectral localization. We develop a transport theory that connects the dominant propagation site to the maximal random-walk excursion within an expanding light cone and predicts a universal Lévy-arcsine statistics, in quantitative agreement with experiment. Our results decouple eigenstate localization from transport and establish ENHSL as a new paradigm for wave dynamics.

## Abstract (web summary)

Here, the authors show that erratic non-Hermitian skin localization allows acoustic waves to localize at disorder-dependent bulk positions while retaining ballistic transport, linking this behavior to the extreme-value statistics of random walks.

## Introduction

Wave localization is a unifying phenomenon across physics, shaping transport in systems ranging from electrons in solids to light, sound, and other engineered media. The most celebrated example, Anderson localization^1–3^, arises when static disorder disrupts an underlying periodic structure, producing exponentially localized eigenstates and strongly inhibited motion. Even in special models where the localization length becomes unbounded, transport remains at most sub-ballistic^4–6^, and transitions to extended phases proceed via mobility edges through critical, fractal states^3,7^.

Non-Hermitian systems^8^—featuring gain, loss, or effective nonreciprocal couplings^9^—have recently expanded the landscape of localization and transport phenomena. A paradigmatic example is the non-Hermitian skin effect (NHSE)^10–25^, where asymmetric couplings cause an extensive set of bulk modes to accumulate at boundaries, overturning conventional bulk-boundary correspondence. In disordered non-Hermitian systems, Anderson localization also arises^26–35^; yet, unlike Hermitian settings, wave propagation can persist even when all eigenstates remain exponentially localized. This counterintuitive transport, driven by stochastic lifetimes of localized modes^33,36–39^, generates jump-like dynamics and universal sub-ballistic spreading.

Despite these developments, Anderson localization—whether Hermitian or non-Hermitian—has traditionally been associated with two universal traits: exponentially localized eigenstates whose centers fluctuate across disorder realizations, and the suppression of ballistic transport. These principles have long shaped our understanding of how disorder constrains wave propagation. While it is widely acknowledged that, in both Hermitian and non-Hermitian systems, the complete spatial localization of eigenmodes in the bulk (dubbed spectral localization) is typically associated with dynamical localization—or, at most, sub-ballistic transport—a fundamental open question is whether ballistic transport can coexist with spectral localization, which would represent a highly unexpected and unconventional regime.

Here we experimentally demonstrate a fundamentally different localization regime—erratic non-Hermitian skin localization (ENHSL)—that emerges in globally reciprocal non-Hermitian lattices with disorder^40^ at a topological phase transition point^41^. ENHSL produces spectral localization in the form of macroscopic, highly irregular bulk localization peaks whose positions vary unpredictably across disorder realizations, distinct from both Anderson localization and the NHSE. The resulting spatial profiles show subexponential decay and reflect stochastic interfaces governed by the order statistics of a symmetric random walk^40^. Here we show that ENHSL constitutes a universal disorder-induced localization mechanism that persists across a broad class of reciprocal non-Hermitian systems, largely independent of the microscopic details of their implementation. Using the Hatano–Nelson chain with a fluctuating imaginary gauge field as a paradigmatic model, we experimentally demonstrate that this unusual form of localization can coexist with ballistic transport.

Realizing ENHSL experimentally requires tunable non-Hermitian couplings, controllable disorder over many realizations, global reciprocity, and direct access to both spectral and dynamical observables. Active acoustic lattices meet these requirements, offering tunable couplings via tunable electroacoustic feedback, flexible disorder implementation, and high-precision wave-field measurements^35,42–48^. Recent active acoustic platforms have further demonstrated simultaneous control of nonreciprocal coupling amplitudes and phases, providing the essential ingredients for implementing disordered imaginary gauge fields and probing ENHSL. Following and extending full-response-matrix/Green’s-function-based spectroscopy developed in circuit and acoustic non-Hermitian systems^14,46,48,49^, we further develop complementary time-resolved measurements to directly observe wave-packet propagation, enabling a unified spectral-and-dynamical characterization of ENHSL on the same acoustic platform.

Using this platform to implement a disordered Hatano–Nelson model, we observe the defining signatures of ENHSL: large-scale, sample-specific bulk peaks with widely fluctuating positions across disorder realizations. These measurements constitute the experimental realization of ENHSL and demonstrate that this localization regime is experimentally accessible, robust, and tunable. Beyond spectral signatures, we develop and test a theoretical model for ENHSL transport, revealing that ensemble-averaged dynamics remain ballistic despite strong spectral localization. Excitations are drawn toward the site where the symmetric random-walk landscape attains its maximal excursion within the light cone; the distribution of this dominant site follows the Lévy-arcsine law. This establishes a universal stochastic principle governing transport in disordered non-Hermitian systems and overturns the long-standing belief that strong disorder and spectral (eigenmode) localization must suppress ballistic motion.

## Results

### Model and conceptual overview

**Fig. 1:**
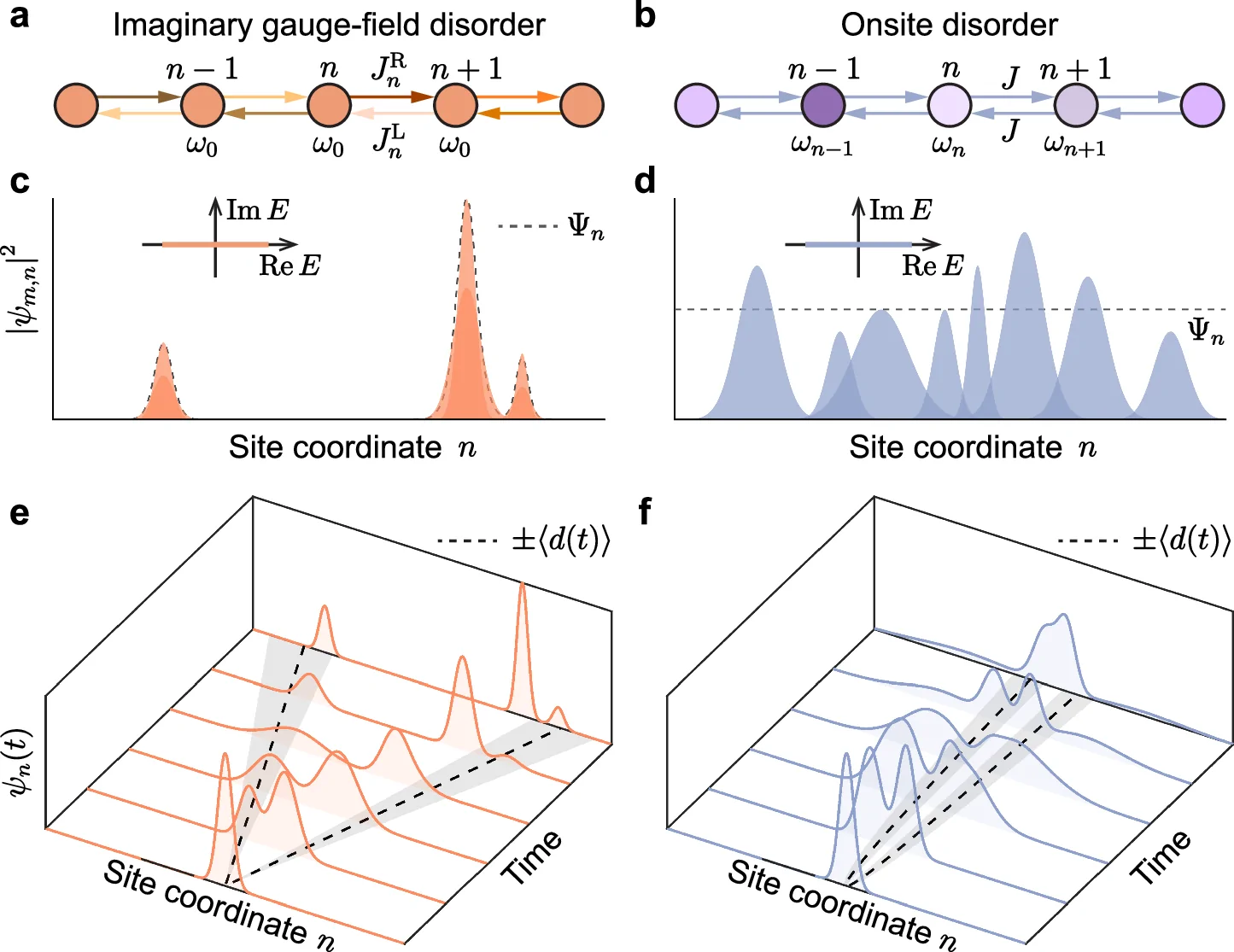
Conceptual comparison between ENHSL and Hermitian Anderson localization. **a** Tight-binding schematic of a globally reciprocal non-Hermitian chain with disordered imaginary gauge fields (hopping disorder) under OBC, which is the physical geometry considered in experiments. Sites share a uniform onsite potential (*ω*_0_), while the link colors indicate spatially fluctuating nonreciprocal hoppings ($\,\,{J}_{n}^{{{\rm{L/R}}}}$). **b** Hermitian Anderson chain with onsite disorder (site colors, *ω*_*n*_) and Hermitian uniform hoppings (*J*). **c** Spectral signatures of ENHSL. The inset sketches the complex spectrum (for either PBC or OBC), which are almost coincident and real under global reciprocity; in the thermodynamic limit, the PBC and OBC spectra coincide. The main panel illustrates a typical ENHSL eigenstate profile for a single representative disorder realization, featuring a dominant bulk peak accompanied by two weaker satellite peaks; the dashed curve indicates the summed eigenstate intensity *Ψ*_*n*_ = ∑_*m*_∣*ψ*_*m*,*n*_∣^2^, which is dominated by the same peak structure. The relative heights and left-right imbalance of these peaks are realization-specific features caused by the particular random-walk landscape *X*_*n*_. **d** Spectral signatures of Anderson localization: many exponentially localized eigenstates centered at different sites, yielding a relatively featureless mode-summed profile *Ψ*_*n*_ (dashed line). **e** Dynamical response in ENHSL following a bulk-centered excitation. The 3D traces show the spatiotemporal evolution of the wave-packet envelope *ψ*_*n*_(*t*) for the same type of single representative disorder realization. Dashed guidelines show the symmetric ensemble-averaged spreading envelope, *n* = ± 〈*d*(*t*)〉 (gray shading: realization-to-realization fluctuations), highlighting the ballistic transport. **f** Dynamical response in the Anderson case, where the wave packet remains dynamically localized, and the ensemble-averaged spreading saturates at long times (dashed guideline).

We consider a paradigmatic model of non-Hermitian disordered systems displaying a topological phase transition, the one-dimensional (1D) Hatano–Nelson chain^26^ with spatially disordered imaginary gauge fields *h*_*n*_^40,41^ comprising *N* sites under either open (OBC) or periodic (PBC) boundary conditions, as sketched in Fig. 1a. In the tight-binding description, the right/left hoppings on link *n* are

1 $${J}_{n}^{{{\rm{R}}}}=J\,{e}^{{h}_{n}},\qquad {J}_{n}^{{{\rm{L}}}}=J\,{e}^{-{h}_{n}},$$

where {*h*_*n*_} are independent random variables drawn from a distribution *f*(*h*) with a mean value $\overline{h}$. The system displays the characteristic NHSE with biased bulk transport when the average gauge field $\overline{h}=(1/N){\sum }_{n}{h}_{n}$ does not vanish, with a topological phase transition at $\overline{h}=0$ signaled by the abrupt change of a topological winding number^34,41^. The critical point $\overline{h}=0$ corresponds to a globally reciprocal system, where the NHSE disappears and a new form of localization, dubbed ENHSL, emerges^40^. A convenient way to represent the gauge disorder is via the cumulative gauge field (random-walk landscape)

2 $${X}_{n}={\sum }_{\ell=0}^{n-1}{h}_{\ell },$$

which maps the link disorder to a random walk in the site coordinate *n*.

In a globally nonreciprocal model ($\overline{h}\ne 0$) the random walk is biased, and one observes the usual features of the NHSE^41^: high sensitivity of the energy spectrum on the boundary conditions (PBC vs OBC), non-vanishing topological winding number, exponential localization of the eigenstates at the lattice edges under OBC with a non-vanishing Lyapunov exponent $\overline{h}$, and biased (unidirectional) transport in the bulk of the lattice. Here we focus our attention on the topological phase transition point $\overline{h}=0$, corresponding to a globally reciprocal lattice and unbiased (symmetric) random walk *X*_*n*_. Typically, we sample *h*_*n*_ from a uniform distribution ${h}_{n} \sim {{\mathcal{U}}}(-\Delta h,\Delta h)$. At such a topological phase transition point, the new form of localization, ENHSL^40^, is observed. Here, eigenstate localization is no longer exponential because of the vanishing of the Lyapunov exponent, and large excursions of *X*_*n*_ give rise to stochastic interfaces that seed macroscopic localization structures through local skin localization. This new form of localization, distinct from both Anderson localization and non-Hermitian skin localization, is not specific to the stochastic Hatano–Nelson model considered in previous works^40^: as shown in Methods, it is a rather universal phenomenon that can be observed in a broad class of globally reciprocal disordered lattices involving long-range hopping and multiple bands, and not restricted to one-dimensional models. A natural and fully open question is whether the spectral localization inherent to the ENHSL, i.e., the erratic spatial localization of all Hamiltonian eigenstates in the bulk, permits any form of transport, or instead fully suppresses it. Although spectral localization is typically linked to dynamical localization or sub-ballistic transport^4–6,33,36–38^, we show here that the extremal statistics of *X*_*n*_ can, quite unexpectedly, enable on average ballistic transport.

To emphasize the genuinely new features of ENHSL, Fig. 1 compares this globally reciprocal non-Hermitian model (Fig. 1a) with a conventional Hermitian (Anderson) disordered chain (Fig. 1b). In the globally reciprocal disordered Hatano–Nelson chain, ENHSL appears in each realization as macroscopic localization peaks at irregular bulk positions, with all eigenstates displaying subexponential localization. A schematic eigenstate profile is shown in Fig. 1c: a dominant bulk peak is typically accompanied by a few weaker satellite peaks, producing a strongly nonuniform mode-summed intensity *Ψ*_*n*_ = ∑_*m*_∣*ψ*_*m*,*n*_∣^2^, which is itself dominated by the same peak structure. Here, *ψ*_*m*,*n*_ is the amplitude of the *m*th (right) eigenstate at site *n* and normalized such that ${\sum }_{n}{\left\vert {\psi }_{m,n}\right\vert }^{2}=1$. A second defining spectral property is that the spectrum is real, and remains essentially insensitive to boundary conditions: switching between open (OBC) and periodic (PBC) boundary conditions does not induce the qualitative spectral reshaping characteristic of the conventional NHSE. For comparison, Fig. 1d sketches the Anderson-localized situation.

A major finding is that the dynamical response provides the sharpest contrast between ENHSL and Anderson localization. Figure 1e illustrates the ENHSL dynamics following a bulk-centered excitation. In a single disorder realization (3D traces), the wave packet does not simply spread and then freeze, nor does it become pinned to a static defect-like site. Instead, the dynamics is dominated by an attraction toward a realization-dependent bulk region associated with the dominant excursion of the random-walk landscape *X*_*n*_ within the finite-time light cone of the underlying disorder-free chain. Accordingly, the wave-packet develops a pronounced peak that is strong, macroscopic, and highly sample-specific. Most importantly, ENHSL exhibits an ensemble-level transport behavior that is counterintuitive from the viewpoint of conventional disorder physics. The ensemble statistics reveal that the spreading distance can remain ballistic on average, which is schematically indicated in Fig. 1e. In other words, ENHSL fully decouples spectral eigenstate localization from transport suppression, allowing ballistic spreading on average. Figure 1f summarizes the Anderson-localized dynamical benchmark, where the wave packet becomes dynamically localized. Ensemble averaging does not restore ballistic motion; instead, it further reinforces the picture of inhibited transport typical of Anderson localization.

### Unified spectral-and-dynamical measurement protocol

We experimentally realize the globally reciprocal Hatano–Nelson model in an engineered acoustic lattice platform^42–48,50–52^. The non-Hermitian hoppings are implemented by programmable electroacoustic feedback between neighboring acoustic resonators, while spatial disorder is introduced by prescribing the link asymmetry sequence {*h*_*n*_}. Different disorder realizations are generated by programming distinct sequences {*h*_*n*_}, implemented through calibrated gain and phase settings in the amplifiers and phase shifters (see Methods). A key feature of the platform is that spectral and dynamical measurements can be performed on the same lattice and for the same programmed disorder realization, enabling a direct comparison between eigenstate localization and wave-packet transport.

**Fig. 2:**
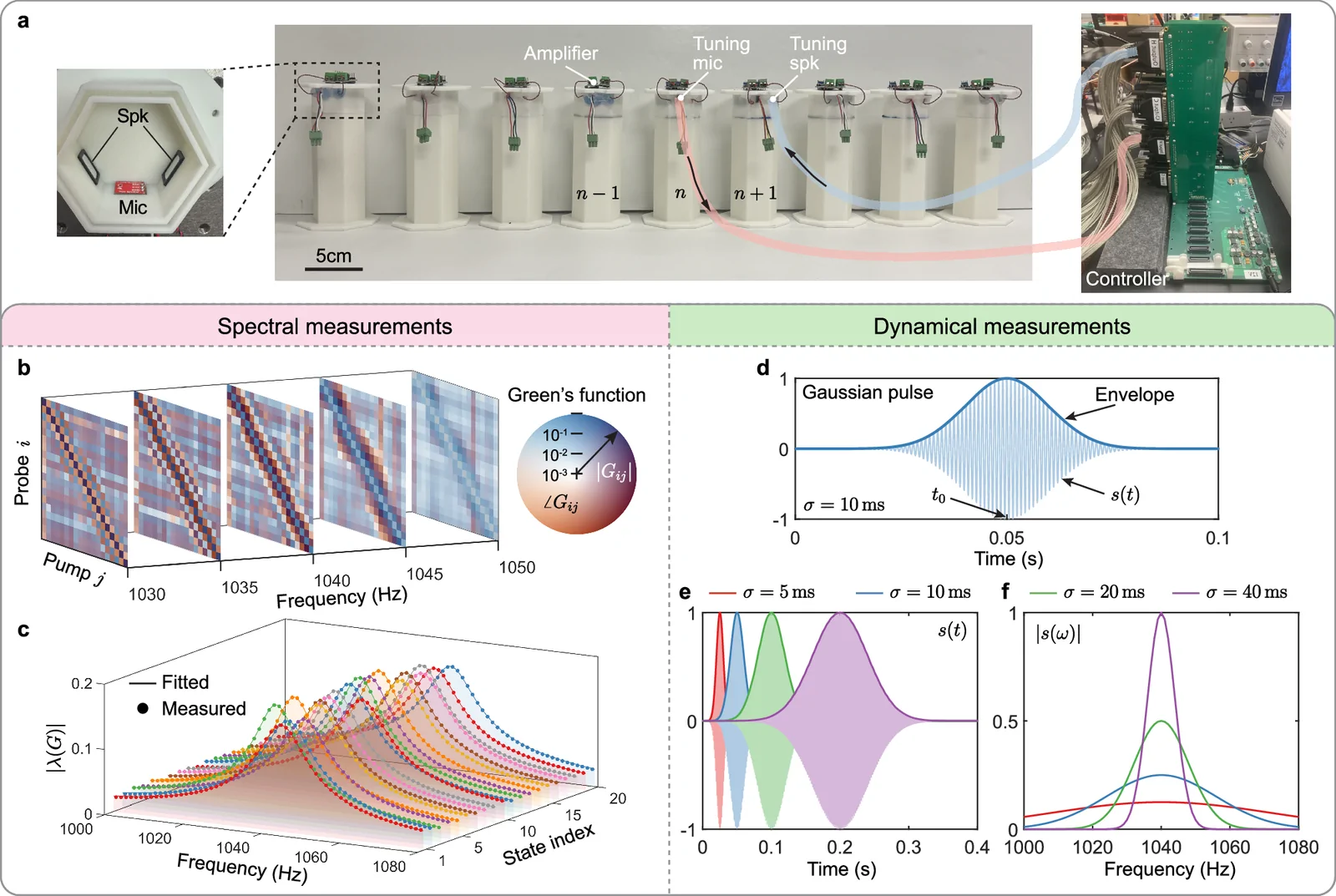
Unified spectral-and-dynamical measurement protocol on the same acoustic lattice. **a** Photograph of the experimental platform: a 1D chain of acoustic resonators (interior view shown at left, containing a microphone and two loudspeakers) coupled by electroacoustic feedback implemented with external amplifiers and phase shifters integrated in a multi-channel controller (right). Here, a representative hopping from site *n* to site *n* + 1 is illustrated. **b**, **c** Green’s -function-based spectroscopy for full spectral reconstruction. **b** Site-by-site excitation (pump *j*) together with full spatial readout (probe *i*) yields the frequency-resolved response (Green’s -function) matrix *G*_*ij*_(*ω*). Shown is an example lattice of size *N* = 20 under OBC (*ω*_0_ = 1040 Hz − 6*i* Hz, *J* = 1.5 Hz, *Δh* = 2), with *G*_*ij*_(*ω*) visualized at several representative frequencies. The inset color wheel indicates the visualization scheme: hue encodes the phase *∠G*_*ij*_, while brightness encodes the magnitude ∣*G*_*ij*_∣ (log-scaled). **c** Extraction of complex eigenenergies from the measured Green’s function. Dots show the measured magnitudes of the eigenvalues *λ*_*m*_(*ω*) of *G*(*ω*) as functions of frequency, and solid curves show fits to a single-pole form *λ*_*m*_(*ω*) ≃ *A*_*m*_/(*ω* − *E*_*m*_), from which the complex eigenenergies *E*_*m*_ are obtained. **d**–**f** Time-resolved wave-packet measurements using the same lattice. **d** Gaussian-modulated tone burst *s*(*t*) centered at ${{\rm{Re}}}({\omega }_{0})=1040\,{{\rm{Hz}}}$, illustrating the carrier and the Gaussian envelope (width *σ*) with temporal center *t*_0_ = 5*σ*. **e**, **f** Excitation waveforms *s*(*t*) (**e**) and their spectra ∣*s*(*ω*)∣ (**f**) for four representative envelope widths *σ* = 5, 10, 20, and 40 ms.

In the spectral measurements, we perform site-by-site steady-state excitation at site *j* and record the complex response at all probe sites *i*, thereby obtaining the frequency-resolved Green’s-function matrix *G*_*ij*_(*ω*) over the relevant spectral band (Fig. 2b). By diagonalizing the measured matrix *G*(*ω*) and fitting the resulting eigenvalue trajectories to the pole form *λ*_*m*_(*ω*) ≃ *A*_*m*_/(*ω* − *E*_*m*_), we extract the complex eigenenergies *E*_*m*_. The corresponding eigenstates *ψ*_*m*,*n*_ are obtained from the eigenvectors of *G*(*ω*) near each resonance (Fig. 2c). This full-matrix reconstruction enables us to evaluate mode-resolved quantities such as the inverse participation ratio (IPR) and the mode-summed intensity *Ψ*_*n*_ for the same disorder realization (see Methods and ref. ^48^ for details).

In the dynamical measurements, we independently excite the central site with a Gaussian-modulated tone burst *s*(*t*) centered at the single-cavity resonance frequency ${{\rm{Re}}}({\omega }_{0})$ (Fig. 2d),

3 $$s(t)=\exp \left[-\frac{{(t-{t}_{0})}^{2}}{2{\sigma }^{2}}\right]\exp \left[-i\,2\pi {{\rm{Re}}}({\omega }_{0})t\right],$$

where *t*_0_ is the pulse center time and *ω*_0_ denotes the complex resonance frequency of a single cavity, with ${{\rm{Im}}}({\omega }_{0})$ accounting for intrinsic loss. The pulse width *σ* is chosen to balance two requirements: it must be large enough to inject sufficient energy above the noise floor, but small enough to provide a spectral bandwidth covering the relevant lattice modes (Fig. 2e, f). The time-resolved acoustic signals are recorded simultaneously at all sites using synchronized data-acquisition modules. After bandpass filtering around the carrier frequency at ${{\rm{Re}}}({\omega }_{0})$, we extract the analytic-signal envelope and use it as the site-resolved wave-packet amplitude *ψ*_*n*_(*t*). At each time *t*, the measured envelope is normalized by its instantaneous norm to obtain

4 $${P}_{n}(t)=\frac{| {\psi }_{n}(t){| }^{2}}{{\sum }_{{n}^{{\prime} }}| {\psi }_{{n}^{{\prime} }}(t){| }^{2}},$$

which is used to compute the spreading distance *d*(*t*) and dominant site *n*_0_(*t*) (see Methods). Thus, the spectral reconstruction and the time-domain propagation measurements provide complementary but directly comparable information on the same ENHSL realization.

### Experimental observation of ENHSL spectral signatures

**Fig. 3:**
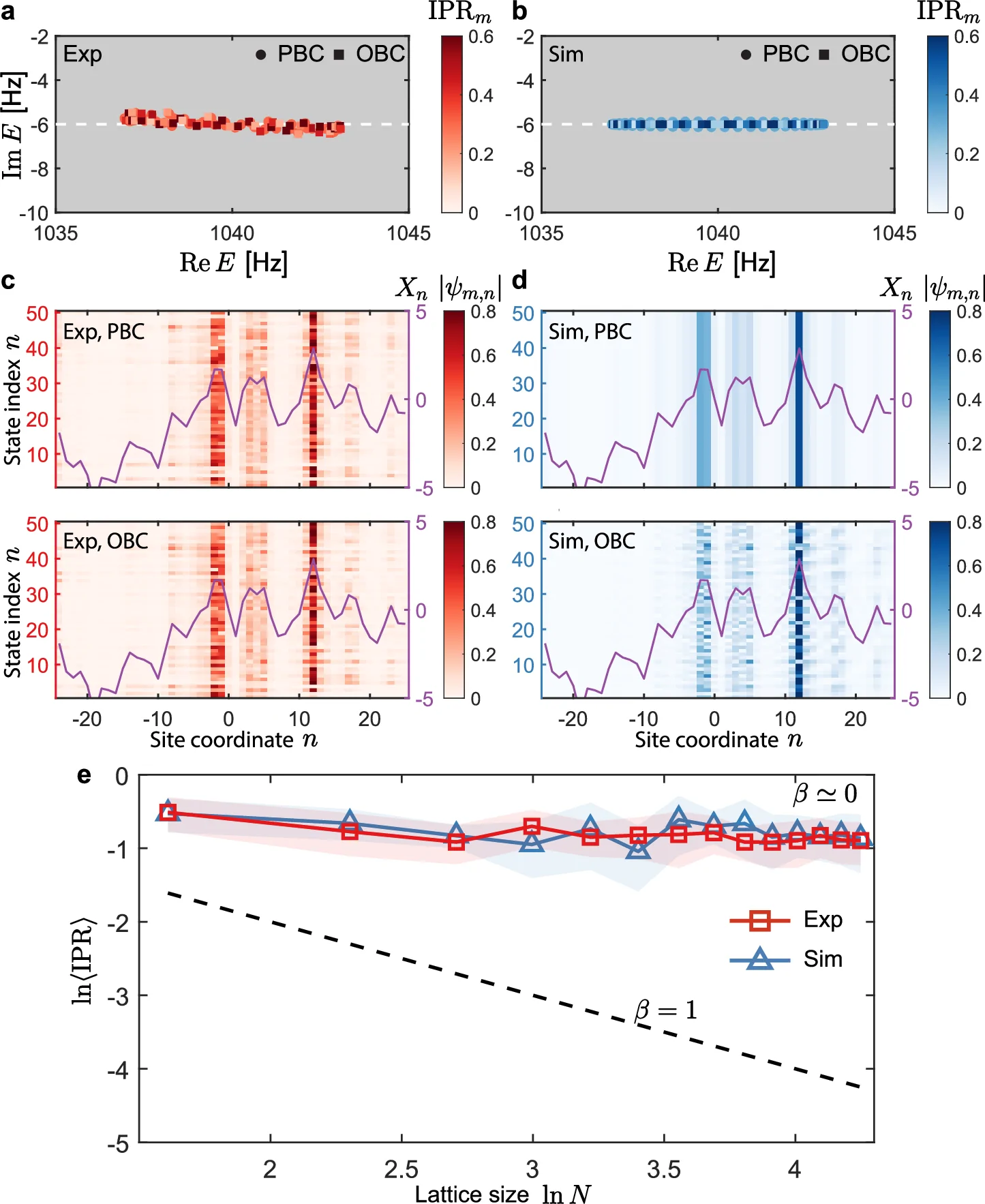
Spectral signatures of ENHSL, comparing experiments and tight-binding simulations for a chain of size *N* = 50 with *ω*_0_ = 1040 Hz − 6*i* Hz, *J* = 1.5 Hz, and *Δh* = 2. **a**, **b** Complex spectra for a representative realization under both PBC (Periodic Boundary Condition) and OBC (Open Boundary Condition). Circles (squares) denote PBC (OBC) energies, and colors encode IPR_*m*_. The dashed horizontal line marks the nominal uniform background-loss level (−6 Hz) used as a reference. **c**, **d**, Spatial distributions of all eigenstates under PBC (top) and OBC (bottom) for experiment (**c**) and simulation (**d**). Heat maps show the eigenstate amplitudes ∣*ψ*_*m*,*n*_∣ as functions of eigenstate index *m* and site coordinate *n*. The overlaid purple curve (right axis) shows the random-walk landscape *X*_*n*_ associated with the imaginary gauge-field disorder, highlighting that prominent localization peaks occur near large excursions of *X*_*n*_. **e** Scaling of localization with system size under OBC. Symbols show the ensemble-averaged 〈IPR〉 for *N* = 5, 10, 15, ..., 70 (each *N* averaged over *R* = 8 disorder realizations), comparing experiment and simulation; shaded bands indicate the realization-to-realization spread (standard deviation). The dashed guideline (*β* = 1) indicates the extended-state scaling IPR ∝ *N*^−1^. A fit to 〈IPR〉 ∝ *N*^−*β*^ yields *β* ≃ 0, consistent with size-independent localization in ENHSL.

We first experimentally observe the spectral features of ENHSL under both PBC and OBC. Figure 3a, b compares experimental and tight-binding simulated spectra for a representative realization, showing good agreement. Despite locally nonreciprocal hoppings, the spectrum remains nearly real up to an overall uniform loss shift (−6 Hz) for both PBC and OBC. Figure 3c, d shows the spatial distributions of the eigenstates ∣*ψ*_*m*,*n*_∣, with the random-walk landscape *X*_*n*_ overlaid (right axis). ENHSL manifests as macroscopic localization peaks whose positions correlate with large excursions of *X*_*n*_, and which persist and are only weakly modified when switching between PBC and OBC. This weak boundary sensitivity contrasts sharply with the conventional NHSE in clean nonreciprocal lattices, where switching boundary conditions qualitatively reshapes both spectrum and localization^48^. From Fig. 3b, d, we see that all eigenstates have the same IPR under PBC, whereas under OBC, a small subset of states shows noticeable deviations. Nevertheless, all eigenstates remain strongly localized under both boundary conditions.

To quantify the system-size dependence, we performed experiments for varying lattice size *N* = 5 to *N* = 70  in steps of 5 under OBC. For each lattice size, we measured *R* = 8 disorder realizations (see Supplementary Information for representative realizations). For each realization, we compute the mode-averaged IPR, ${\overline{{{\rm{IPR}}}}}_{r}={N}^{-1}{\sum }_{m=1}^{N}{{{\rm{IPR}}}}_{m}$, and then average over realizations to obtain $\langle {{\rm{IPR}}}\rangle={R}^{-1}\mathop{\sum }_{r=1}^{R}{\overline{{{\rm{IPR}}}}}_{r}$. Figure 3e presents the averaged IPR versus chain length *N*, with good agreement between experiment and simulation. We characterize the size scaling by fitting 〈IPR〉 ∝ *N*^−*β*^, where *β* is an effective scaling exponent. For extended and localized states, *β* takes the values 1 and 0, respectively, while 0 < *β* < 1 indicates fractal states. As shown in Fig. 3e, $\ln \langle {{\rm{IPR}}}\rangle$ is approximately independent of lnN, yielding *β* ≃ 0. This experimental observation confirms the strong spectral localization of eigenstates in ENHSL, although the localization is subexponential due to the vanishing of the Lyapunov exponent^40^.

### Theoretical description of ENHSL dynamics

The spectral formulation of ENHSL describes eigenstate localization via the random-walk landscape *X*_*n*_ induced by the stochastic imaginary gauge field. Here, we present a complementary dynamical perspective. This viewpoint is essential for uncovering the fact that ballistic transport survives despite the macroscopic, erratically positioned bulk localization peaks of ENHSL—a phenomenon that fundamentally departs from both Anderson localization and conventional NHSE.

To reveal the single-particle dynamics, we apply the non-unitary gauge transformation ${\psi }_{n}(t)={\phi }_{n}(t)\,\exp ({X}_{n})$, which maps the system onto a uniform Hermitian nearest-neighbor chain with hopping *J* (see Methods). For a single-site initial excitation at *n* = 0, the Hermitian solution is given by ${\phi }_{n}(t)={(-i)}^{n}{{{\mathcal{J}}}}_{n}(2Jt)$, where ${{{\mathcal{J}}}}_{n}(\cdot )$ is the Bessel function of first kind. Transforming back to the original variables, the normalized occupation probability profile at site *n* and time *t* is

5 $${P}_{n}(t)=\frac{| {\psi }_{n}(t){| }^{2}}{{\sum }_{{n}^{{\prime} }}| {\psi }_{{n}^{{\prime} }}(t){| }^{2}}=\frac{{{{\mathcal{J}}}}_{n}^{2}(2Jt)\exp \left(2{X}_{n}\right)}{{\sum }_{{n}^{{\prime} }}{{{\mathcal{J}}}}_{{n}^{{\prime} }}^{2}(2Jt)\exp \left(2{X}_{{n}^{{\prime} }}\right)}.$$

We quantify transport in a single realization using the second moment $d(t)=\sqrt{{\sum }_{n}{n}^{2}{P}_{n}(t)}$. In the disorder-free limit *h*_*n*_ = 0 (hence *X*_*n*_ = 0), one recovers ballistic spreading $d(t)=\sqrt{2}\,Jt$. Therefore, the disorder-free ballistic spreading is retained in the underlying propagator, while the stochastic gauge factors reshape where intensity accumulates. As a result, the finite-time intensity profile can be viewed as a Hermitian ballistic core modulated by an envelope $\exp \left(2{X}_{n}\right)$, which locally amplifies (*X*_*n*_ > 0) or suppresses (*X*_*n*_ < 0) intensity. This separation between intrinsic (Hermitian) propagation and disorder-induced amplification does not, by itself, explain why ballistic transport can coexist with spectrally localized eigenstates. A naïve “excitation-funneling” picture^16^—in which the wave packet is simply attracted toward the global maximum of *X*_*n*_—would incorrectly predict full dynamical localization, since the intensity would then accumulate near a single disorder-selected site. Crucially, however, this intuition is flawed: although *X*_*n*_ has a well-defined global maximum, its location can lie arbitrarily far from the initial excitation, and the distance to this maximum fluctuates wildly between disorder realizations. Thus, determining how far the wave packet can travel, and with what statistics, is a highly nontrivial problem. The correct analysis necessarily involves the extreme-value statistics of random walks, rather than typical disorder fluctuations. To clarify this essential point, let us observe that at finite time *t*, the Bessel kernel confines the dynamics with a super-exponential localization to a light-cone-like accessible interval ∣*n*∣ ≲ *n*_LB_(*t*), with a linear-in-time Lieb-Robinson (LB) bound *n*_LB_(*t*) ≃ 2*Jt*. Within this interval, ENHSL dynamics is controlled by an extremal statistic: the wave packet is predominantly localized around the dominant site

$${n}_{0}(t)=\arg {\mathop{\max }_{n}}{P}_{n}(t),$$

which follows the location where *X*_*n*_ attains its largest excursion within the light cone. This mechanism is qualitatively different from the standard picture of transport in disordered systems, where motion is shaped by typical fluctuations. Here, the dynamics is governed by extreme-value fluctuations of a random walk, revealing that transport at the ENHSL transition point is dictated by rare, sample-specific random-walk excursions.

As *X*_*n*_ is a symmetric random walk, the position of its maximum over a finite interval exhibits a universal limiting distribution according to the Sparre–Anderson theorem^53–56^. Consequently, for sufficiently large *t*, the empirical distribution of *n*_0_(*t*) approaches the Lévy-arcsine law mapped to the interval [−*n*_LB_(*t*), *n*_LB_(*t*)] (derivation in Methods),

6 $${P}_{{{\rm{arc}}}}({n}_{0},t)=\frac{1}{\pi \sqrt{[{n}_{{{\rm{LB}}}}(t)-{n}_{0}][{n}_{0}+{n}_{{{\rm{LB}}}}(t)]}},\qquad | {n}_{0}| < {n}_{{{\rm{LB}}}}(t).$$

Equation (6)—which we both derive theoretically and verify experimentally—constitutes a central result of this work. It demonstrates that the dominant-propagation site obeys the universal Lévy-arcsine distribution, establishing an unexpected bridge between non-Hermitian transport and classical extreme-value statistics of random walks. To our knowledge, such a connection has neither been predicted nor observed previously in any wave-transport setting.

Equation (6) predicts an enhanced probability of finding the dominant site near the light-cone edges, ∣*n*_0_∣ ~ *n*_LB_(*t*). Furthermore, by using Eq. (6), the second moment averaged over all locations can be approximated as $\langle d(t)\rangle \simeq \sqrt{{\sum }_{n}{n}^{2}{P}_{{{\rm{arc}}}}({n}_{0}=n,t)}=\sqrt{2}Jt$, which clearly shows the ballistic scaling. Thus, despite the strongly localized and erratically positioned eigenstates characterizing ENHSL, the ensemble-averaged transport remains rigorously ballistic—a nontrivial and striking consequence of the arcsine-governed extremal dynamics.

### Experimental observation of single-realization dynamics

**Fig. 4:**
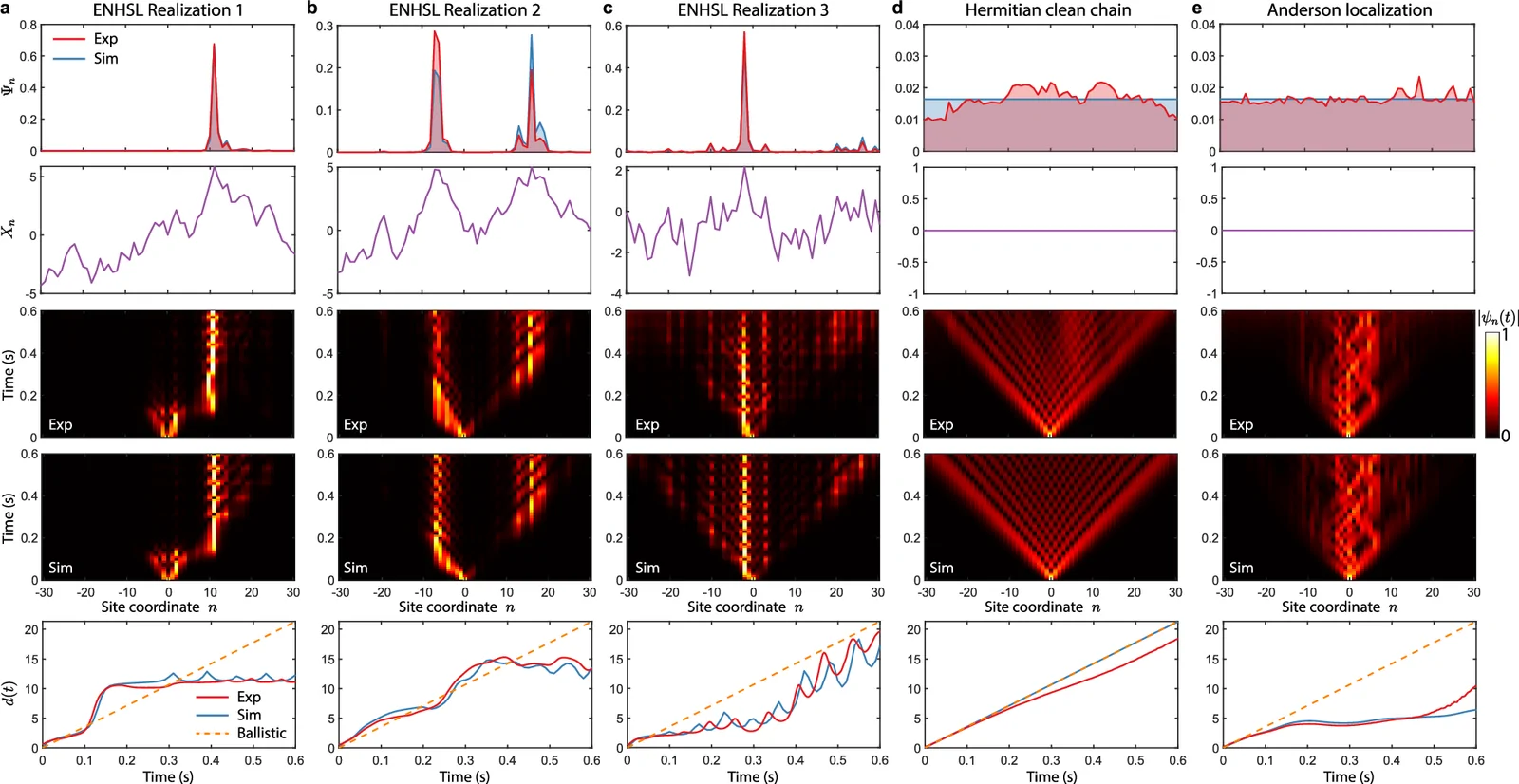
Experimental observation of wave-packet propagation following excitation at the central site (*n* = 0) in a chain of length *N* = 61 under OBC. The parameters set in experiments are *ω*_0_ = 1040 Hz − 1.75*i* Hz, *J* = 4 Hz, and *Δh* = 1.6. **a**–**c** Three representative disorder realizations of the ENHSL. **d** Disorder-free Hermitian chain (clean reference). **e** Hermitian chain with onsite disorder showing Anderson localization (reference). For each panel, rows from top to bottom show: (i) the spatial profile of the eigenstate intensity summed over all states, *Ψ*_*n*_ = ∑_*m*_∣*ψ*_*m*,*n*_∣^2^; (ii) the random-walk landscape *X*_*n*_; (iii) the experimentally measured spatiotemporal evolution of the acoustic pressure envelope $\left\vert {\psi }_{n}(t)\right\vert$; (iv) the corresponding tight-binding simulation; and (v) the spreading distance *d*(*t*) extracted from experiment (red solid curves) and simulation (blue solid curves), with the ballistic law shown for comparison (orange dashed).

Guided by the dynamical theory, we first experimentally observe the extremal-selection mechanism at the level of individual disorder realizations. Figures 4a–c present three representative realizations for ENHSL dynamics (see Supplementary Information for more realizations), compared with two reference cases: (Fig. 4d) a clean Hermitian chain (ballistic spreading benchmark) and (Fig. 4e) a disordered Hermitian chain (Anderson localization benchmark). For each ENHSL realization (Fig. 4a–c), the mode-summed eigenstate intensity ${\Psi }_{n}={\sum }_{m}{\left\vert {\psi }_{m,n}\right\vert }^{2}$ exhibits a prominent macroscopic peak (often accompanied by a few weaker satellite peaks). This peak structure is fundamentally different from the Anderson reference (Fig. 4e), where the sum over many exponentially localized eigenstates with random centers yields a comparatively featureless *Ψ*_*n*_. In ENHSL, the random-walk profile *X*_*n*_ shown in the second row of each panel acts as the organizing structure: prominent spectral peaks emerge near its large excursions, consistent with the spectral picture.

The third row of Fig. 4 shows the measured spatiotemporal evolution $\left\vert {\psi }_{n}(t)\right\vert$ following excitation at the central site. The corresponding simulation results are presented in the fourth row, showing good agreement with the experiment for all configurations. In each ENHSL realization, $\left\vert {\psi }_{n}(t)\right\vert$ develops a pronounced localization peak at an irregular bulk position, in agreement with the theoretical picture that dynamics is drawn toward a dominant excursion of *X*_*n*_. Importantly, this localization peak does not simply coincide with a fixed Anderson-like center; rather, the region of maximal intensity is selected by the disorder realization and can evolve in time within the expanding light cone interval. In several realizations, the dominant intensity ridge in the (*n*, *t*) plane reveals a slow drift of the peak position as time increases, reflecting the fact that *n*_0_(*t*) is defined by an extremal statistic on an expanding interval $\left(-{n}_{{{\rm{LB}}}}(t),{n}_{{{\rm{LB}}}}(t)\right)$. In contrast, the Hermitian disorder-free reference (Fig. 4d) exhibits the ballistic spreading pattern, while the Anderson reference (Fig. 4e) shows rapid confinement of $\left\vert {\psi }_{n}(t)\right\vert$ near the initial excitation site, consistent with dynamical localization. Although the realizations in Fig. 4a, c show similar dominant peaks in the mode-summed intensity *Ψ*(*n*), their time-domain responses differ because Fig. 4c contains weak satellite structures on the right side of the lattice. These realization-specific satellite peaks, generated by fluctuations of the zero-mean imaginary gauge field, provide additional transient weight away from the main peak and make the spatiotemporal profile appear more extended. Such differences are a direct manifestation of the erratic nature of ENHSL and do not affect the ensemble-level ballistic transport discussed below.

To quantify spreading, we extract a spreading distance *d*(*t*) from $\left\vert {\psi }_{n}(t)\right\vert$ and compare experiment and tight-binding simulations (bottom row of Fig. 4). For ENHSL realizations, *d*(*t*) can grow fast and saturate slowly (Fig. 4a) or grow slower than the ballistic reference (Fig. 4c), or exhibit intermediate behaviours (Fig. 4b). These representative realizations show distinct evolution patterns for ENHSL depending on the disorder realization, reflecting strong sample-to-sample fluctuations. For reference, the clean Hermitian chain (Fig. 4d) shows *d*(*t*) growing almost linearly with time, while the Anderson reference (Fig. 4e) shows *d*(*t*) saturating quickly. We note that the spreading distance *d*(*t*) can vary substantially between individual ENHSL realizations. This is expected because *d*(*t*) is a second moment-type observable and is therefore sensitive to weak satellite structures at large ∣*n*∣. For example, realization 3 contains additional transient weight away from the dominant peak, producing a more extended and oscillatory *d*(*t*) than realizations 1 and 2. Such sample-specific differences reflect the erratic nature of ENHSL dynamics and are quantitatively reproduced by simulations; the robust ballistic behavior emerges only after ensemble averaging, as shown below.

### Experimental observation of ensemble dynamics and Lévy-arcsine law

**Fig. 5:**
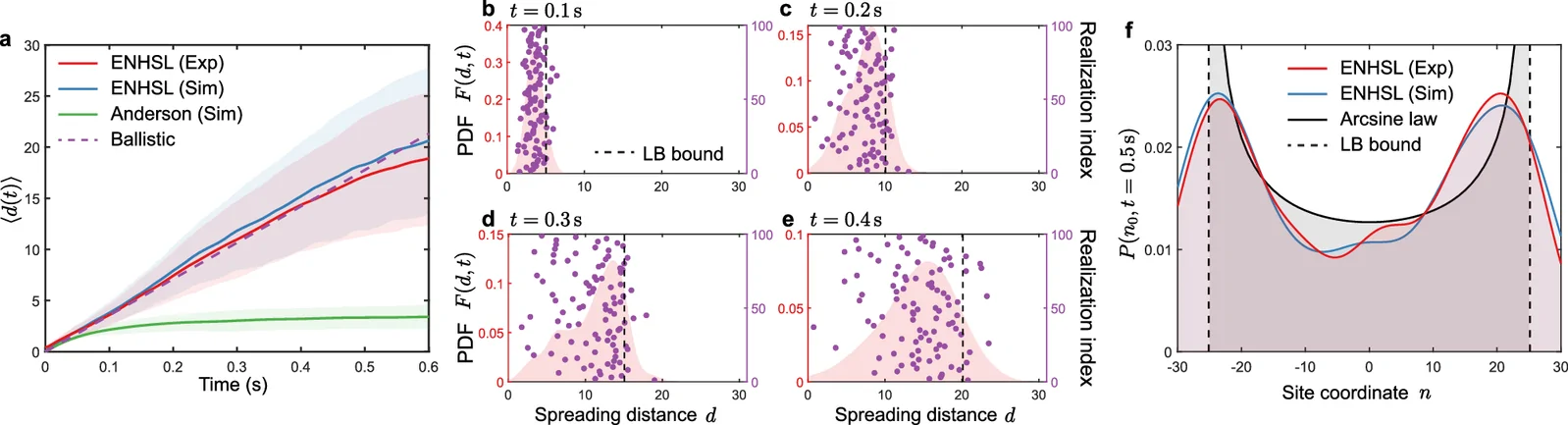
Ensemble statistics of wave-packet spreading for the ENHSL (OBC, *N* = 61), obtained from *R* = 100 disorder realizations. The parameters set in experiments are *ω*_0_ = 1040 Hz − 1.75*i* Hz, *J* = 4 Hz, and *Δh* = 1.6. **a** Ensemble-averaged spreading distance 〈*d*(*t*)〉 from experiments and tight-binding simulations, compared with the ballistic scaling of a clean Hermitian chain and with a Hermitian Anderson-localized reference (shown by simulations, exhibiting saturation). **b**–**e** Distributions of the spreading distance at fixed times *t* = 0.1, 0.2, 0.3, and 0.4s, respectively. In each panel, purple markers indicate the values *d*^(*r*)^(*t*) from individual realizations *r* (realization index shown on the right axis). The shaded curves show the probability density function (PDF) *F*(*d*, *t*) (left axis) estimated from the finite ensemble using a bounded KDE method on the interval (0, 30). The dashed lines denote the Lieb-Robinson (LB) bound, *n*_LB_(*t*) = 2*Jt*. **f**, PDF of the dominant-propagation site, ${n}_{0}(t)=\arg {{\max }_{n}}\left\vert {\psi }_{n}(t)\right\vert$, at *t* = 0.5s, obtained from the same *R* = 100 realizations, and estimated using a bounded KDE method on the interval (−30, 30). Experimental and simulated distributions are compared. The dashed vertical lines indicate the corresponding LB bounds, ±*n*_LB_, and the black curve shows the Lévy-arcsine-law prediction restricted to the interval (−*n*_LB_, *n*_LB_).

We now turn from representative realizations to ensemble-level transport and statistics. Figure 5a shows the ensemble-averaged (*R* = 100 realizations) spreading distance 〈*d*(*t*)〉 extracted from the measured wave packets and compared with tight-binding simulations. The ENHSL data exhibit a robust linear growth over the experimentally accessible time window, consistent with ballistic transport. For comparison, the clean Hermitian chain shows the ballistic scaling, whereas the Anderson-localized reference saturates, reflecting conventional dynamical localization.

Beyond the mean, Fig. 5b–e presents the distributions of *d*(*t*) at several fixed times. To faithfully estimate the probability density from a finite ensemble without boundary artefacts, we employ a bounded kernel density estimation (KDE) on the finite support of *d* (caption of Fig. 5). These panels visualize two important aspects of ENHSL dynamics. First, the distributions are broad and strongly time dependent, reflecting significant realization-to-realization fluctuations. This broadness is expected because the dynamics is governed by extremal statistics of the random walk *X*_*n*_, which naturally produces large sample-to-sample variability. Second, the weight of the distribution shifts to larger *d* as time increases, consistent with the expansion of the accessible light-cone interval. In contrast, the Anderson reference would exhibit distributions that rapidly narrow and remain confined, consistent with saturation of *d*(*t*).

While *d*(*t*) provides a natural bulk measure of spreading, the most discriminating test of the dynamics theory concerns the dominant-site statistics. For each realization, we extract ${n}_{0}(t)=\arg {{\max }_{n}}\left\vert {\psi }_{n}(t)\right\vert$, and obtain its distribution *P*(*n*_0_, *t*). The theory predicts that *n*_0_(*t*) follows the Lévy-arcsine law on the finite interval (−*n*_LB_(*t*), *n*_LB_(*t*)), Eq. (6), where the bound *n*_LB_(*t*) = 2*Jt* is fixed by the independently known coupling *J*. Figure 5f shows the measured *P*(*n*_0_, *t*) at *t* = 0.5s (correspondingly, *n*_LB_ = 25) and compares it with both simulation and the arcsine prediction. The measured distribution is strongly nonuniform and enhanced toward the edges of the accessible interval, as expected from the arcsine law. This agreement constitutes a stringent verification because it tests the full functional form rather than just a scaling trend, and hinges on an extremal statistic that is explicitly connected to the underlying random walk.

The arcsine-law verification also provides an explanation for ballistic ensemble transport. A symmetric random walk attains its maximum near the boundaries of a finite interval with high probability, and therefore the typical magnitude of the maximizer scales as ∣*n*_0_∣~*n*_LB_(*t*). Since *n*_LB_(*t*) ∝ *t*, the typical displacement associated with the dominant peak grows linearly, yielding ballistic scaling for 〈*d*(*t*)〉 even though each realization appears localized at any fixed time.

## Discussion

Our results provide the experimental demonstration of a localization regime in non-Hermitian systems, in which strong, macroscopic localization robustly coexists with ballistic transport. Distinct from both Hermitian Anderson localization—where spectral localization is inseparable from dynamical arrest—and from the conventional NHSE—where bulk modes accumulate exponentially at boundaries—ENHSL establishes a fundamentally different paradigm: globally reciprocal non-Hermiticity and disorder jointly generate realization-dependent bulk localization peaks with subexponential profiles, while the associated wave-packet dynamics remains transport preserving on average. A central outcome of our work is the identification and verification of an extremal-statistics mechanism for transport. By combining full spectral reconstruction with time-resolved measurements on the same platform, we directly connect the dominant-propagation site to the largest excursion of a symmetric random-walk landscape within a finite-time light cone, and we confirm the resulting universal Lévy-arcsine statistics. Beyond identifying a new form of wave localization, our findings open new directions in the study of complex wave dynamics and reveal a broadly applicable mechanism relevant to quantum and classical waves alike—from condensed matter and photonics to acoustics, metamaterials and other complex media. More generally, ENHSL highlights a route toward engineering stochastic yet controllable wave confinement with predictable ensemble transport, and it motivates future studies of how extremal statistics, topology, interactions, or higher dimensions reshape the relation between localization and propagation in complex non-Hermitian systems.

## Methods

### General theoretical framework of the ENHSL

To clarify the concept and universality of ENHSL, we consider a general *Hermitian* 1D lattice with an arbitrary number *M* of orbitals per unit cell and arbitrary long-range hopping. Let *ϕ*_*n*_ denote the wave-function amplitude at lattice site *n* (including orbital indices), and write the clean Hermitian eigenvalue problem as

7 $$E{\phi }_{n}={\sum}_{l}{H}_{n,l}{\phi }_{l},$$

where ${H}_{n,l}={H}_{l,n}^{*}$ encodes all intra-cell and inter-cell hoppings, possibly of arbitrarily long range. Owing to discrete spatial translation invariance, the eigenvectors of Eq. (7) are Bloch waves ${\phi }_{n} \sim \exp (ikn)$ with real energies defined by the dispersion relations *E* = *E*_*α*_(*k*) of the *M* bands, where *α* = 1, 2, ..., *M* is the band index and  − *π*≤*k* < *π* is the Bloch wave number that varies in the first Brillouin zone. We now impose a site-dependent (fluctuating) imaginary gauge field *h*_*n*_, inducing the non-unitary transformation

8 $${\phi }_{n}={\psi }_{n}\exp (-{X}_{n}),$$

where *h*_*n*_ are independent and equally-distributed random variables and ${X}_{n}={\sum }_{l=1}^{n}{h}_{l}$ describes a classical random walk on the lattice. This non-unitary transformation generates an effective non-Hermitian Hamiltonian

9 $${\tilde{H}}_{n,l}={H}_{n,l}\exp ({X}_{n}-{X}_{l})$$

with $E{\psi }_{n}={\sum }_{l}{\tilde{H}}_{n,l}{\psi }_{l}$. Because the differences (*X*_*n*_ − *X*_*l*_) fluctuate spatially, Eq. (9) describes a strongly disordered non-Hermitian lattice even when the underlying Hermitian model is perfectly clean.

Let us now consider the spectral problem of the non-Hermitian lattice with Hamiltonian $\tilde{H}$. While, for the original clean Hermitian Hamiltonian *H*, the energy spectrum is entirely real and insensitive to boundary conditions (except for the possible appearance of accidental edge states under OBC), this is no longer the case for the companion non-Hermitian Hamiltonian $\tilde{H}$ due to the NHSE. Under OBC (*ψ*_0_ = *ψ*_*N*+1_ = 0), the energy spectra of *H* and $\tilde{H}$ are identical. This follows from the fact that the non-unitary transformation Eq. (8) is invariant under OBC, modifies only the eigenvectors, leaving the spectrum invariant. Conversely, under PBC (*ψ*_*N*+1_ = *ψ*_1_), the spectrum of $\tilde{H}$ is, in general, complex. It can be obtained from the spectrum of *H*, *E*_*α*_(*k*), by deforming the Brillouin zone and analytically continuing *k* into the complex plane according to $k\to k-i\overline{h}$, where

10 $$\overline{h}={\mathop{\lim }_{N\to \infty }}\frac{1}{N}{\sum }_{n=1}^{N}{h}_{n},$$

is the mean imaginary gauge field, capturing the global non-reciprocity of the disordered system. Indeed, the boundary condition *ψ*_*N*+1_ = *ψ*_1_ implies ${\phi }_{N+1}={\phi }_{1}\exp ({X}_{1}-{X}_{N+1})$, which, in the large-*N* limit, is satisfied by ${\phi }_{n} \sim \exp (ikn-\overline{h}n)$ with $kN=2\pi \ {{\rm{mod}}}\ 2\pi$. This yields the complex energy spectrum ${E}_{\alpha }(k-i\overline{h})$. When $\overline{h}\ne 0$, the system exhibits the conventional NHSE: the energy spectrum depends sensitively on boundary conditions (OBC versus PBC), and under OBC a macroscopic accumulation of eigenstates at one edge occurs. In this regime, the PBC spectrum is characterized by a non-vanishing topological winding number^34,41^, which determines the collapse direction of the skin modes. Reversing the orientation of the winding, i.e., changing the sign of $\overline{h}$, reverses the direction of this accumulation.

The key structural condition for ENHSL is global reciprocity, $\overline{h}=0$, corresponding to a topological phase transition point where the winding number changes sign. In this case, the imaginary gauge field has no extensive bias, and the net nonreciprocal drift vanishes in the thermodynamic limit *N* → *∞*. The condition $\overline{h}=0$ prevents the formation of a conventional NHSE, in which all eigenstates accumulate at a boundary due to a finite global drift. Under global reciprocity, the imaginary gauge deformation does not induce an extensive complex energy shift, the topological winding number vanishes, and the energy spectrum remains real and becomes asymptotically independent of boundary conditions:

11 $${{\lim }_{N\to \infty }}{\sigma }_{{{\rm{OBC}}}}(\tilde{H})={{\lim }_{N\to \infty }}{\sigma }_{{{\rm{PBC}}}}(\tilde{H})=\sigma (H)\subset {\mathbb{R}}.$$

Intuitively, local non-Hermiticity from random factors $\exp ({X}_{n})$ does not sum to a macroscopic drift and therefore cannot complexify the bulk spectrum under PBC. Even though the global drift vanishes, the random fluctuations of the effective hopping asymmetries can remain strong and spatially erratic. These fluctuations generate position-dependent nonreciprocal biases that vary across the lattice and originate from the extreme-value statistical properties of the symmetric random walk *X*_*n*_. As a consequence, individual eigenstates experience local “patches” of NHSE pointing in different directions, leading to disorder-dependent, sample-specific localization centers distributed throughout the bulk, without any macroscopic accumulation at a boundary. This localization mechanism is fundamentally different from Anderson localization; rather than arising from destructive interference, it originates from the non-unitary gauge transformation in Eq. (8) and is rooted in the extreme-value statistics of symmetric random walks^40^. This behavior constitutes the hallmark of ENHSL.

The above analysis demonstrates that ENHSL is a universal phenomenon in disordered non-Hermitian lattices generated via imaginary gauge deformations of Hermitian parent models, provided that global reciprocity Eq. (10) holds. This remains true regardless of the number of internal degrees of freedom *M*, the hopping range (including long-range couplings), the details of the clean band structure, or the dimensionality (the reasoning extends naturally to 2D and 3D). Thus, ENHSL is a generic feature of globally reciprocal non-Hermitian systems with spatially varying imaginary gauge fields. A prototypical and simplest model exhibiting ENHSL is the fluctuating Hatano–Nelson model, which is used in our experiments.

### Details of the dynamics theory of ENHSL

In the following, we focus on the one-dimensional Hatano–Nelson chain with a stochastic imaginary gauge field *h*_*n*_ defined on each link, with hopping amplitudes as given in Eq. (1). However, the analysis can be naturally extended to more general lattices with fluctuating imaginary gauge fields, constructed via the general procedure outlined above (i.e., as non-Hermitian companions of clean Hermitian lattices, possibly including multiple bands and long-range hopping). The key result is that, on average, transport remains ballistic and is not affected by the disordered gauge fields, despite the fact that all eigenstates of the Hamiltonian are localized.

#### Model and evolution equation

In the single-particle sector, the dynamical equations for the (non-normalized) probability amplitudes *ψ*_*n*_(*t*) satisfy

12 $$i\frac{d{\psi }_{n}}{dt}={J}_{n}^{{{\rm{L}}}}{\psi }_{n+1}+{J}_{n-1}^{{{\rm{R}}}}{\psi }_{n-1}=J\left({e}^{-{h}_{n}}{\psi }_{n+1}+{e}^{{h}_{n-1}}{\psi }_{n-1}\right),$$

with either OBC (*ψ*_0_ = *ψ*_*N*+1_ = 0) or PBC (*ψ*_*n*+*N*_ = *ψ*_*n*_). Note that under OBC, boundary effects play no role in wave packet expansion provided the excitation starts in the bulk, and the wave packet does not reach the edges within the observation time.

#### Random-walk landscape and non-unitary gauge transformation

The cumulative gauge field (random-walk landscape) is defined as Eq. (2), which maps the sequence {*h*_*n*_} to a discrete-time symmetric random walk in *n*. Introduce the non-unitary gauge transformation

13 $${\psi }_{n}(t)={\phi }_{n}(t)\,\exp ({X}_{n})\equiv {\phi }_{n}(t)\,{u}_{n},\qquad {u}_{n}=\exp ({X}_{n}).$$

Substituting Eq. (13) into Eq. (12) yields the uniform Hermitian transport equation

14 $$i\frac{d{\phi }_{n}}{dt}=J({\phi }_{n+1}+{\phi }_{n-1}).$$

For a single-site initial excitation at *n* = 0, the Hermitian solution is

15 $${\phi }_{n}(t)={(-i)}^{n}{{{\mathcal{J}}}}_{n}(2Jt),$$

where ${{{\mathcal{J}}}}_{n}(\cdot )$ is the Bessel function of the first kind. Therefore,

16 $$| {\psi }_{n}(t){| }^{2}={{{\mathcal{J}}}}_{n}^{2}(2Jt)\,\exp (2{X}_{n})={{{\mathcal{J}}}}_{n}^{2}(2Jt)\,{u}_{n}^{2}.$$

In the experiment we analyze the normalized occupation probability profile

17 $${P}_{n}(t)=\frac{| {\psi }_{n}(t){| }^{2}}{{\sum }_{{n}^{{\prime} }}| {\psi }_{{n}^{{\prime} }}(t){| }^{2}}=\frac{{{{\mathcal{J}}}}_{n}^{2}(2Jt)\exp (2{X}_{n})}{{\sum }_{{n}^{{\prime} }}{{{\mathcal{J}}}}_{{n}^{{\prime} }}^{2}(2Jt)\exp (2{X}_{{n}^{{\prime} }})}.$$

Equation (17) makes explicit the central structure. The temporal spreading of the wave packet resembles the standard Hermitian Bessel profile, modulated by the random envelope ${u}_{n}^{2}=\exp (2{X}_{n})$, which locally amplifies (*X*_*n*_ > 0) or suppresses (*X*_*n*_ < 0) intensity. The excitation is statistically drawn toward the dominant excursion (local maxima) of *X*_*n*_ within the dynamically accessible interval, producing strong localization peaks in each realization.

#### Light-cone bound and unit convention

The Bessel kernel ${{{\mathcal{J}}}}_{n}^{2}(2Jt)$ decays rapidly, with tails falling faster than exponentially outside the interval ∣*n*∣ ≳ 2*Jt*, effectively defining a Lieb-Robinson bound—or light cone—for nearest-neighbor hopping:

18 $$| n| \lesssim {n}_{{{\rm{LB}}}}(t),\qquad {n}_{{{\rm{LB}}}}(t)\simeq 2Jt.$$

If the hopping is reported in angular-frequency units (rad/s), Eq. (18) is used directly. If the hopping is reported in Hz (cycles/s), one should convert *J*_*ω*_ = 2*πJ*_Hz_ and write

19 $${n}_{{{\rm{LB}}}}(t)\simeq 2{J}_{\omega }t=2(2\pi {J}_{{{\rm{Hz}}}})\,t.$$

In this work, all hoppings and onsite potentials are reported in Hz, so Eq. (19) is used.

#### Second-moment spreading observable

The evolution of the wave packet’s spatial spread in a single realization of disorder is quantified using the second moment:

20 $$d(t)=\sqrt{{\sum}_{n}{n}^{2}{P}_{n}(t)}=\sqrt{\frac{{\sum }_{n}{n}^{2}{{{\mathcal{J}}}}_{n}^{2}(2Jt)\exp (2{X}_{n})}{{\sum }_{n}{{{\mathcal{J}}}}_{n}^{2}(2Jt)\exp (2{X}_{n})}}.$$

In the disorder-free limit *h*_*n*_ = 0 (hence *X*_*n*_ = 0), one recovers ballistic spreading $d(t)=\sqrt{2}\,Jt$.

#### Lévy-arcsine law for dominant-site distribution

Let ${u}_{n}=\exp ({X}_{n})$ and consider the thermodynamic limit *N* → *∞*. From the central limit theorem, for large *n* one has

21 $$\frac{1}{\sqrt{2n}}\log \left\vert \frac{{u}_{n}}{{u}_{0}}\right\vert \sim {{\mathcal{N}}}(0,\Delta {h}^{2}),$$

where ${{\mathcal{N}}}(0,\Delta {h}^{2})$ is the normal distribution with zero mean and variance *Δh*^2^, so that typical envelope amplitudes behave as

22 $${u}_{n} \sim \exp \left(-\Delta h\sqrt{n}\right),$$

showing a subexponential but stronger-than-algebraic decay^40^.

For finite time *t*, the Bessel profile ${{{\mathcal{J}}}}_{n}^{2}(2Jt)$ is confined within the light cone ∣*n*∣ < *n*_LB_(*t*) = 2*Jt*, with rapid decay outside. Thus, ∣*ψ*_*n*_(*t*)∣^2^ is predominantly localized around the site *n* = *n*_0_(*t*) where *X*_*n*_ attains its maximum within ( − *n*_LB_(*t*), *n*_LB_(*t*)):

23 $${n}_{0}(t)=\arg {{\max }_{-{n}_{{{\rm{LB}}}} < n < {n}_{{{\rm{LB}}}}}}{X}_{n}.$$

Here *n*_0_ is a random variable with a universal limiting distribution for large *t*, which follows from the Sparre–Andersen theorem^53–56^. This theorem states that many fluctuation properties of symmetric random walks do not depend on the actual distribution *f*(*h*), provided that increments are continuous and the distribution is symmetric about *h* = 0. Assuming that the random walk has independent and identically distributed symmetric ±*h* increments, the probability distribution can be calculated using standard ballot/reflection arguments^55,56^ and reads

24 $$P({n}_{0}=n,t)=\frac{\left(\begin{array}{c}2(n+{n}_{{{\rm{LB}}}})\\ n+{n}_{{{\rm{LB}}}}\end{array}\right)\left(\begin{array}{c}2({n}_{{{\rm{LB}}}}-n)\\ {n}_{{{\rm{LB}}}}-n\end{array}\right)}{\left(\begin{array}{c}4{n}_{{{\rm{LB}}}}\\ 2{n}_{{{\rm{LB}}}}\end{array}\right)},\qquad n=-{n}_{{{\rm{LB}}}}+1,\ldots,{n}_{{{\rm{LB}}}}-1,$$

with *n*_LB_(*t*) = 2*Jt*. Using Stirling’s approximation,

25 $$\left(\begin{array}{c}2n\\ n\end{array}\right) \sim \frac{{4}^{n}}{\sqrt{\pi n}},$$

for large *n*, this reduces to

26 $${P}_{{{\rm{arc}}}}({n}_{0}=n,t) \sim \sqrt{\frac{{n}_{{{\rm{LB}}}}}{\pi ({n}_{{{\rm{LB}}}}-n)({n}_{{{\rm{LB}}}}+n)}}.$$

The resulting distribution of *n*_0_ is the universal Lévy-arcsine law, which indicates an enhanced probability of finding *n*_0_, and thus localization of excitation, near the light-cone edges *n* → ± *n*_LB_ = ±2*Jt*.

#### Second moment averaged over disorder realizations

An approximate expression of the second moment averaged over all locations can be computed as

27 $$\langle d(t)\rangle=\sqrt{{\sum }_{n=-{n}_{{{\rm{LB}}}}}^{{n}_{{{\rm{LB}}}}}{n}^{2}{P}_{{{\rm{arc}}}}({n}_{0}=n,t)}.$$

By setting *n* = *xn*_LB_ with *x* ∈ [−1, 1] and using Eq. (26), in the limit *n*_LB_ → *∞* one has

28 $$\langle d(t)\rangle={n}_{{{\rm{LB}}}}\sqrt{\int_{-1}^{1}{x}^{2}{P}_{0}(x){{\rm{d}}}x}=\sqrt{2}Jt,$$

where ${P}_{0}(x)=\frac{1}{\pi \sqrt{1-{x}^{2}}}$ is the standard arcsine distribution on [−1, 1], and the second moment of *P*_0_(*x*) is known to be 1/2. Equation (28) clearly shows the ballistic scaling of the disorder-averaged second moment. In our experiments, the accessible sites extend to ∣*n*∣ ~30, for which the Stirling-based asymptotic approximation Eq. (25) is already accurate at the percent level.

### Implementation of non-Hermitian acoustic lattices

The non-Hermitian acoustic lattices consist of multiple 3D-printed acoustic cavities (see Supplementary Information for dimensions), each representing a site in the non-Hermitian lattice and operating near its first dipole resonance around 1040 Hz. The tuning of onsite potentials and hoppings is implemented using active components, specifically loudspeaker-microphone (pump-probe) pairs, an audio amplifier (LM386), and a phase shifter integrated in a customized controller (see Supplementary Information for details). The loudspeaker and microphone are positioned at the bottom of each cavity. The microphone captures the acoustic pressure signal, which is processed by the amplifier and phase shifter to adjust its amplitude and phase before being emitted by the loudspeaker in the connected cavity (see ref. ^48^ for details).

The imaginary gauge disorder is implemented by imposing an asymmetry between the right and left hopping magnitudes according to Eq. (1), i.e., ${J}_{n}^{{{\rm{R}}}}/{J}_{n}^{{{\rm{L}}}}=\exp (2{h}_{n})$, while keeping the geometric mean fixed at *J*. Global reciprocity is enforced by choosing sequences {*h*_*n*_} satisfying ∑_*n*_*h*_*n*_ = 0. In practice, the values are calibrated by independent measurements of the effective hoppings (e.g., via two-site characterization, see ref. ^48^).

### Spectral reconstruction via Green’s function measurements

For a chain of *N* sites, we measure the steady-state complex response at all probe sites *i* for each pump site *j* under monochromatic excitation at frequency *ω*. This yields the full-response matrix *G*_*ij*_(*ω*) over a discrete set of drive frequencies spanning the band of interest. The magnitude and phase are obtained by referencing the microphone signals to the excitation signal, allowing coherent extraction of *G*_*ij*_(*ω*). The full matrix acquisition over all pump-probe pairs yields *N*^2^ complex transfer functions per frequency point^48^.

In the effective tight-binding description, the Green’s function satisfies

29 $$G(\omega )={(\omega -H)}^{-1},$$

so the eigenvectors of *G*(*ω*) coincide with those of *H*, and its eigenvalues follow the single-pole form

30 $${\lambda }_{m}(\omega )=\frac{1}{\omega -{E}_{m}}.$$

up to a complex prefactor set by the measurement normalization. We therefore diagonalize *G*(*ω*) for each sampled *ω*, track each eigenvalue branch *λ*_*m*_(*ω*) across frequency, and fit to Eq. (30) to extract *E*_*m*_. This procedure yields both ${{\rm{Re}}}({E}_{m})$ and *ℑ*(*E*_*m*_), allowing direct access to the complex spectrum. Eigenmodes are obtained from the corresponding eigenvectors of *G*(*ω*) evaluated near $\omega \simeq {{\rm{Re}}}({E}_{m})$, where the response is dominated by the target pole. If needed, left eigenvectors can be obtained analogously from the left eigen-decomposition of *G*(*ω*); in the present work, we focus on the mode amplitudes and IPR used in Fig. 3.

### Time-resolved propagation measurements

Time-domain dynamics are initiated by driving the central site (*n* = 0) with a Gaussian-modulated tone burst, Eq. (3). The carrier frequency is set to ${{\rm{Re}}}({\omega }_{0})=1040\,{{\rm{Hz}}}$ (near the band center), and the envelope width is chosen as *σ* = 10 ms following the criteria discussed in the main text (Fig. 2d–f). The driving signal is generated by a multi-channel data-acquisition card (PCIe-6353, National Instruments) and applied to the on-site loudspeaker (CMS-15118D-L100, CUI Devices) through an audio amplifier (LM386, Texas Instruments). The time-domain waveform at each site is recorded simultaneously using MEMS microphones (BOB-19389, SparkFun Electronics) at a sampling rate of 16 kHz, with all channels acquired synchronously.

The recorded pressure signals are digitally bandpass-filtered around the carrier frequency (1010–1070 Hz). The envelope of the signal is taken as $\left\vert {\psi }_{n}(t)\right\vert$. At each time *t*, we normalize *ψ*_*n*_(*t*) by its instantaneous norm, so that ${\sum }_{n}{\left\vert {\psi }_{n}(t)\right\vert }^{2}=1$ for all *t*.

To obtain ensemble statistics, we repeat the measurement for *R* disorder realizations and evaluate ${n}_{0}^{(r)}(t)$ at a fixed evolution time *t* (chosen as large as permitted by avoiding boundary reflections). The empirical distribution is

31 $$\widehat{P}({n}_{0},t)=\frac{1}{R}{\sum }_{r=1}^{R}{\delta }_{{n}_{0},{n}_{0}^{(r)}(t)},$$

where *δ*_*i*,*j*_ is the Kronecker delta. For visualization from a finite ensemble, we further smooth $\widehat{P}({n}_{0},t)$ using a bounded, boundary-corrected Gaussian kernel density estimator on the finite support *n*_0_ ∈ (−*n*_LB_(*t*), *n*_LB_(*t*)), in which the Gaussian kernel centered at each sample is truncated to the interval and renormalized to remove boundary bias; the bandwidth is selected via leave-one-out likelihood cross-validation.

## Supplementary information

Supplementary information

Transparent Peer Review file

## Data Availability

All the data supporting this study are available in the paper and Supplementary Information. Additional data related to this paper are available from the corresponding authors upon request.

## Code availability

The custom codes for this study that support the findings are available from the corresponding authors upon request.
