# Supplementary material for: Observation of erratic non-Hermitian skin localization and transport
Source: Nat Commun. 2026 Aug 21;17:9979. doi: 10.1038/s41467-026-76908-3 (PMC13590536; doi:10.1038/s41467-026-76908-3)
Supplement: Supplementary file 1 [file 41467_2026_76908_MOESM1_ESM.pdf]

Supplementary Information

Observation of Erratic Non-Hermitian Skin Localization and Transport

Jia-Xin Zhong[1,2], Jee Woo Kim[2], Stefano Longhi[3,4,*], and Yun Jing[2,†]

[1]Key Laboratory of Modern Acoustics and Institute of Acoustics, Nanjing University, Nanjing 210093, China

[2]Graduate Program in Acoustics, The Pennsylvania State University, University Park, PA 16802, USA

[3]Dipartimento di Fisica, Politecnico di Milano, Piazza Leonardo da Vinci 32, I-20133 Milano, Italy

[4]IFISC (UIB-CSIC), Instituto de Fisica Interdisciplinary Sistemas Complejos, E-07122 Palma de Mallorca, Spain

Corresponding author: *stefano.longhi@polimi.it; †yqj5201@psu.edu

Contents

Section S1. Extended experimental details 2

Section S2. Extended experimental data for spectral signatures of ENHSL 2

Section S3. Extended experimental data for dynamical observations of ENHSL . . 7

References 8

Section S1. Extended experimental details

The acoustic lattice is constructed using multiple 3D-printed acoustic cavities, each representing a site in the non-Hermitian lattice and operating near its first dipole resonance around 1040 Hz. The cavity dimensions are shown in Fig. S1. The cavities are hexagonal in shape with a side length of 25 mm and a height of 164 cm. Acoustic cavities are interconnected through active pump-probe pairs consisting of a loudspeaker and a microphone, along with an audio amplifier (LM386) and a phase shifter integrated in a customized controller [1]. The tuning of onsite potentials and hoppings is implemented using these active components, as shown in Fig. S1, and detailed in [1]. To summarize, the onsite potential at each site is controlled by a pump-probe pair within the same cavity, while the hopping between adjacent sites is controlled by a pump-probe pair connecting two neighboring cavities. The amplifier and phase shifter allow for independent adjustment of the amplitude and phase of the acoustic signal, enabling precise control over the non-Hermitian parameters of the lattice.

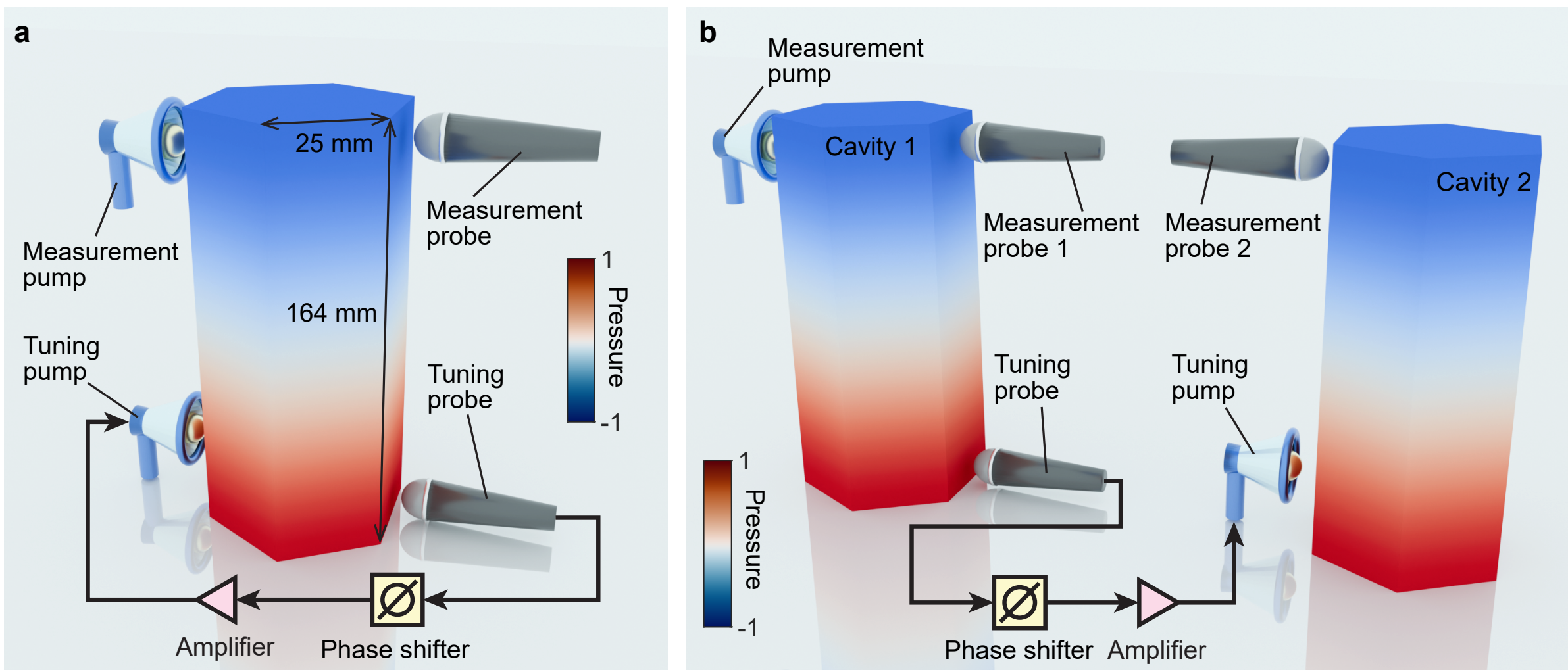

Figure S1: Sketche of the experimental acoustic cavity showing the dimensions and the implementation of tunable (a) onsite potentials and (b) hoppings using active components. Figures were adapted from Ref. [2, Fig. 2].

Section S2. Extended experimental data for spectral signatures of ENHSL

Figures S2–S8 present additional representative experimental data for the spectral signatures of ENHSL, comparing experiments and tight-binding simulations for chain sizes $N = 10, 20, 30, 40, 50, 60,$ and 70. In each figure, we observe good agreement between experimental measurements and tight-binding simulations across all chain sizes. The complex spectra under both periodic boundary conditions (PBC) and open boundary conditions (OBC) exhibit similar patterns, with the inverse participation ratio (IPR) color-coded to indicate localization properties. The spatial distributions of eigenstates under PBC and OBC also show consistent

behavior between experiments and simulations, with the random-walk landscape X_n overlaid for reference. These results validate the theoretical predictions of ENHSL and demonstrate the robustness of the experimental implementation across different system sizes.

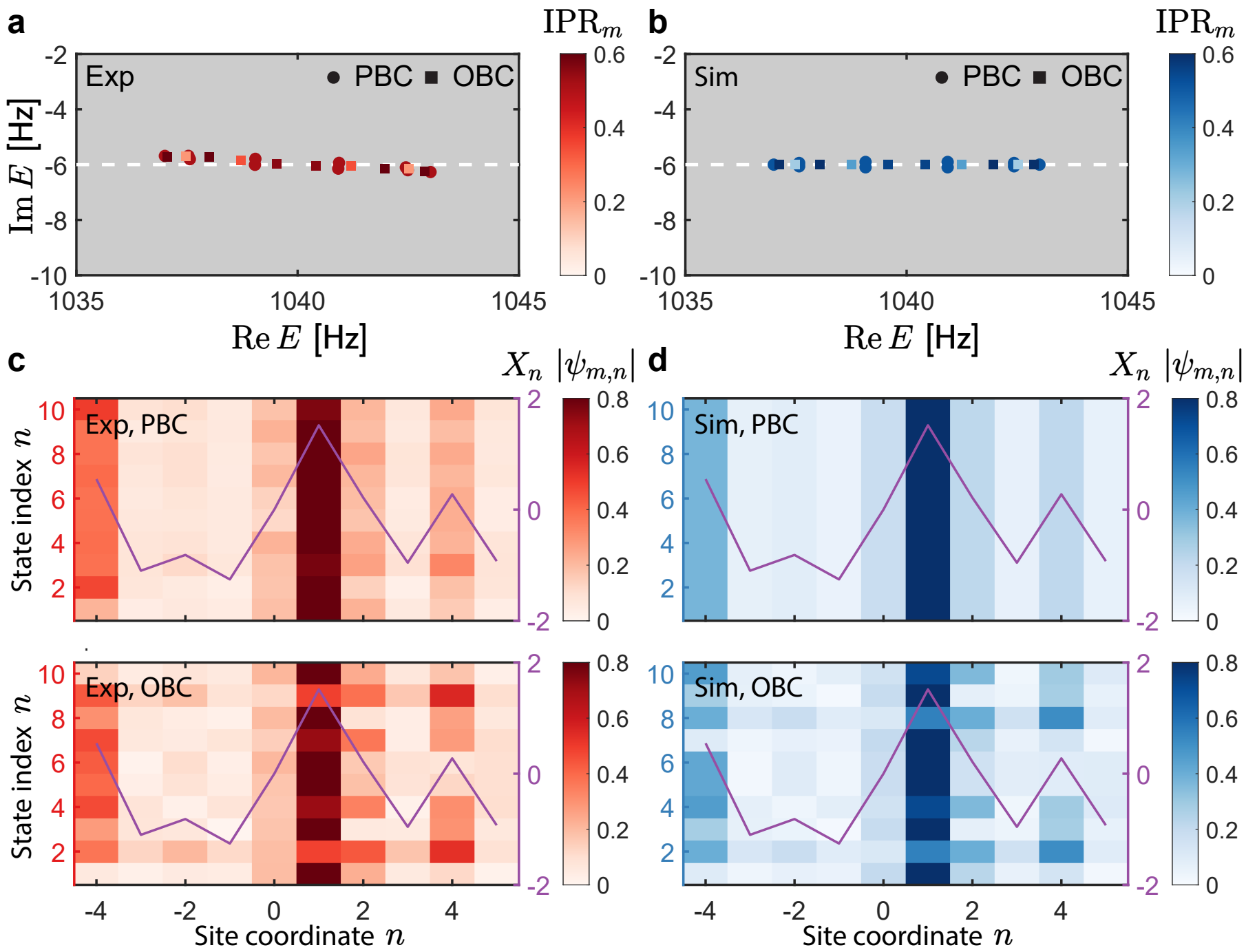

Figure S2: Spectral signatures of ENHSL, comparing experiments and tight-binding simulations for a chain of size $N = 10$ with $\omega_0 = 1040\,\mathrm{Hz} - 6i\,\mathrm{Hz}$, $J = 1.5\,\mathrm{Hz}$, and $\Delta h = 2\,\mathrm{Hz}$. **a,b**, Complex spectra for a representative realization under both PBC and OBC. Circles (squares) denote PBC (OBC) energies, and colors encode IPR_m. **c,d**, Spatial distributions of all eigenstates under PBC (top) and OBC (bottom) for experiment (c) and simulation (d). Heat maps show the eigenstate amplitudes $|\psi_{m,n}|$ as functions of eigenstate index m and site coordinate n. The overlaid purple curve (right axis) shows the random-walk landscape X_n.

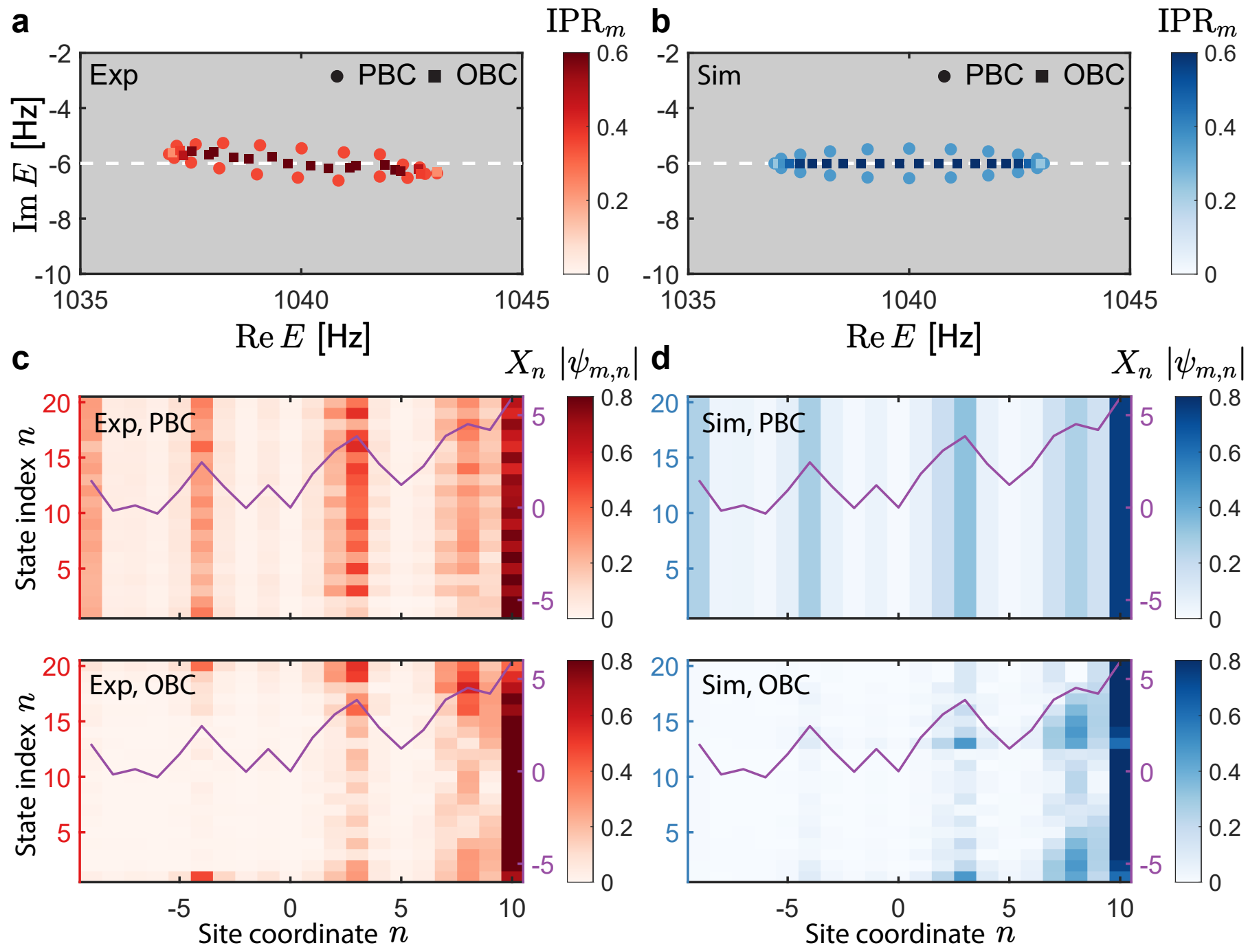

Figure S3: Spectral signatures of ENHSL, comparing experiments and tight-binding simulations for a chain of size $N = 20$ with $\omega_0 = 1040\,\mathrm{Hz} - 6i\,\mathrm{Hz}$, $J = 1.5\,\mathrm{Hz}$, and $\Delta h = 2\,\mathrm{Hz}$. **a,b**, Complex spectra for a representative realization under both PBC and OBC. Circles (squares) denote PBC (OBC) energies, and colors encode IPR_m. **c,d**, Spatial distributions of all eigenstates under PBC (top) and OBC (bottom) for experiment (c) and simulation (d). Heat maps show the eigenstate amplitudes $|\psi_{m,n}|$ as functions of eigenstate index m and site coordinate n. The overlaid purple curve (right axis) shows the random-walk landscape X_n.

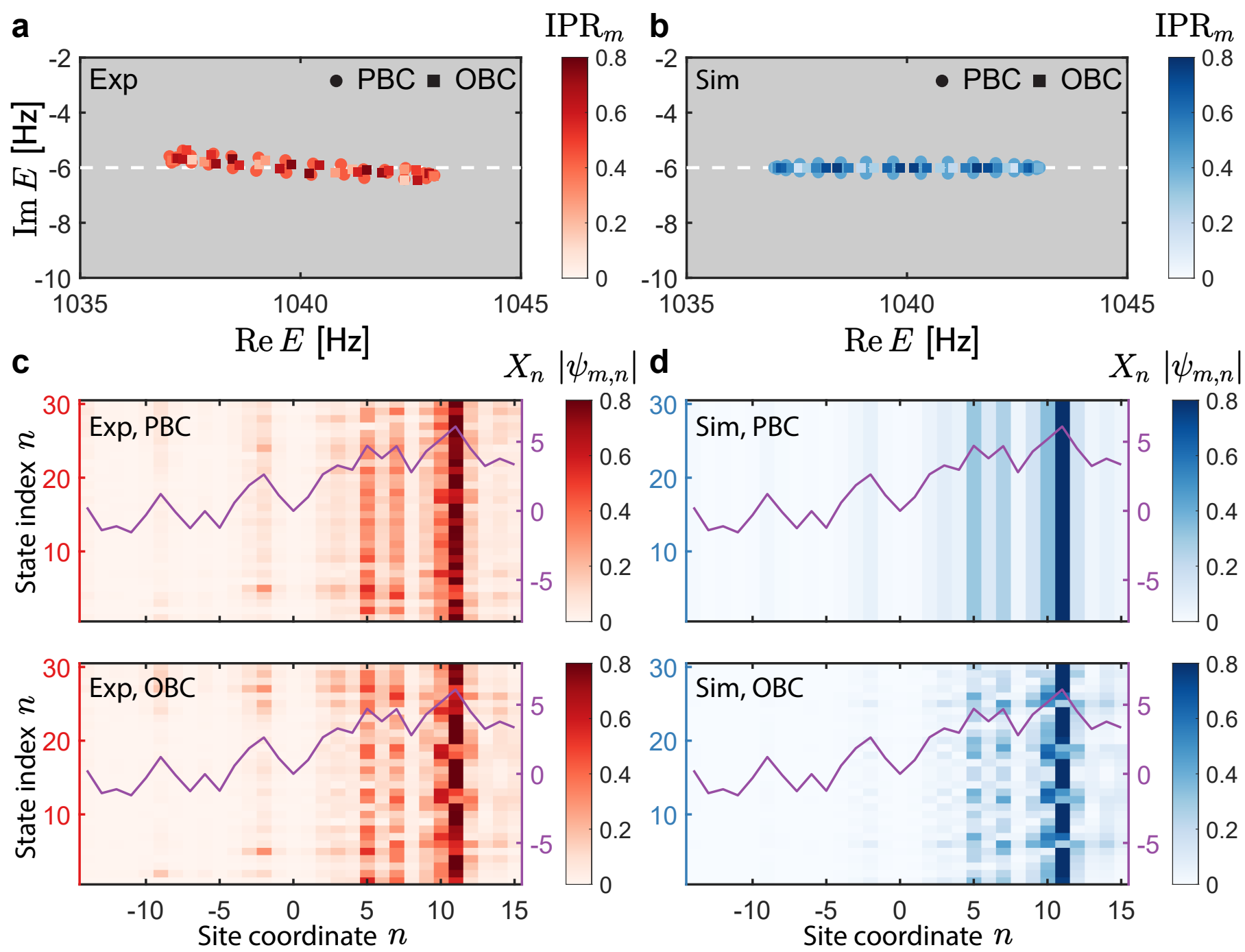

Figure S4: Spectral signatures of ENHSL, comparing experiments and tight-binding simulations for a chain of size $N = 30$ with $\omega_0 = 1040\,\mathrm{Hz} - 6i\,\mathrm{Hz}$, $J = 1.5\,\mathrm{Hz}$, and $\Delta h = 2\,\mathrm{Hz}$. **a,b**, Complex spectra for a representative realization under both PBC and OBC. Circles (squares) denote PBC (OBC) energies, and colors encode IPR_m. **c,d**, Spatial distributions of all eigenstates under PBC (top) and OBC (bottom) for experiment (c) and simulation (d). Heat maps show the eigenstate amplitudes $|\psi_{m,n}|$ as functions of eigenstate index m and site coordinate n. The overlaid purple curve (right axis) shows the random-walk landscape X_n.

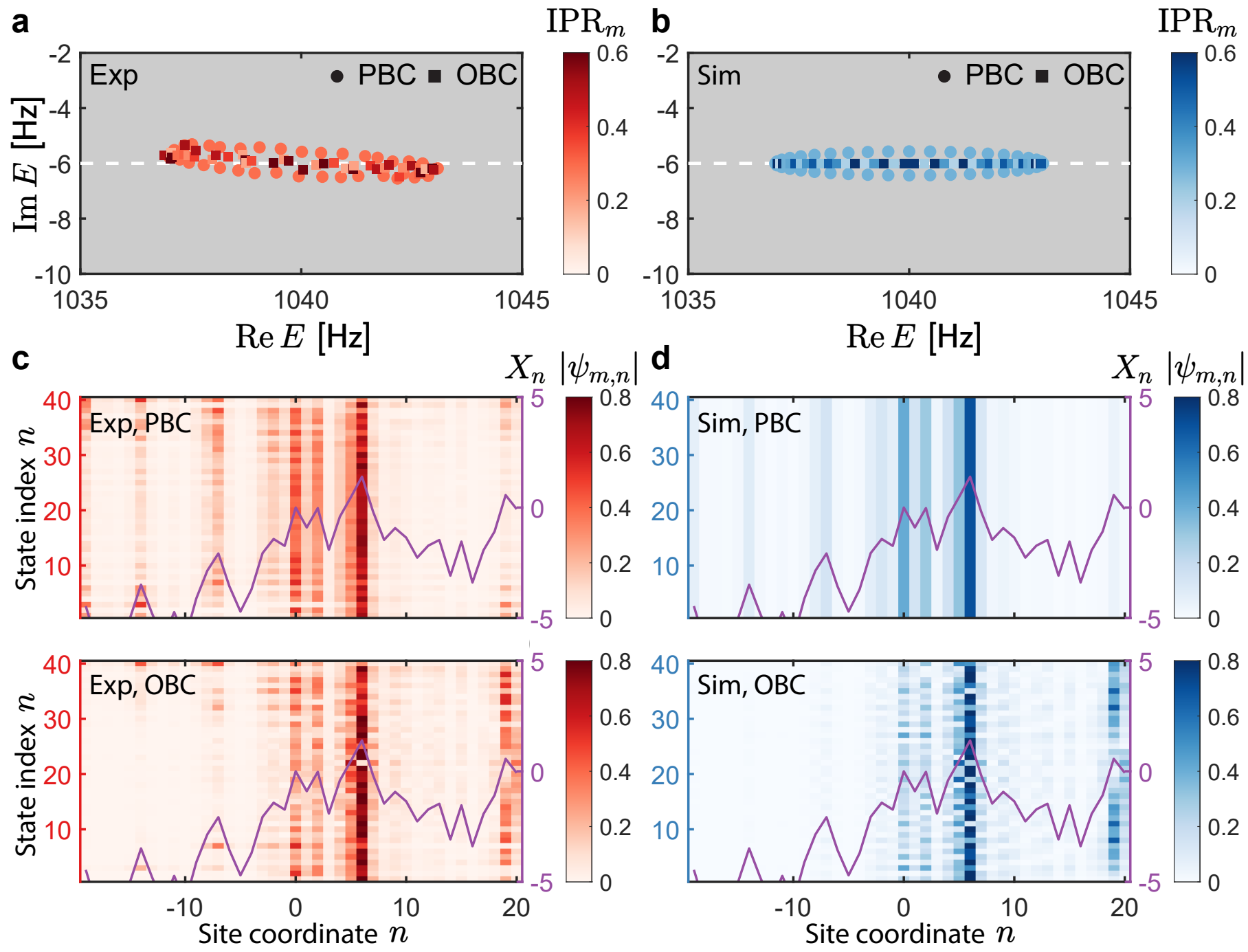

Figure S5: Spectral signatures of ENHSL, comparing experiments and tight-binding simulations for a chain of size $N = 40$ with $\omega_0 = 1040\,\mathrm{Hz} - 6i\,\mathrm{Hz}$, $J = 1.5\,\mathrm{Hz}$, and $\Delta h = 2\,\mathrm{Hz}$. a,b, Complex spectra for a representative realization under both PBC and OBC. Circles (squares) denote PBC (OBC) energies, and colors encode IPR_m. **c,d**, Spatial distributions of all eigenstates under PBC (top) and OBC (bottom) for experiment (c) and simulation (d). Heat maps show the eigenstate amplitudes $|\psi_{m,n}|$ as functions of eigenstate index m and site coordinate n. The overlaid purple curve (right axis) shows the random-walk landscape X_n.

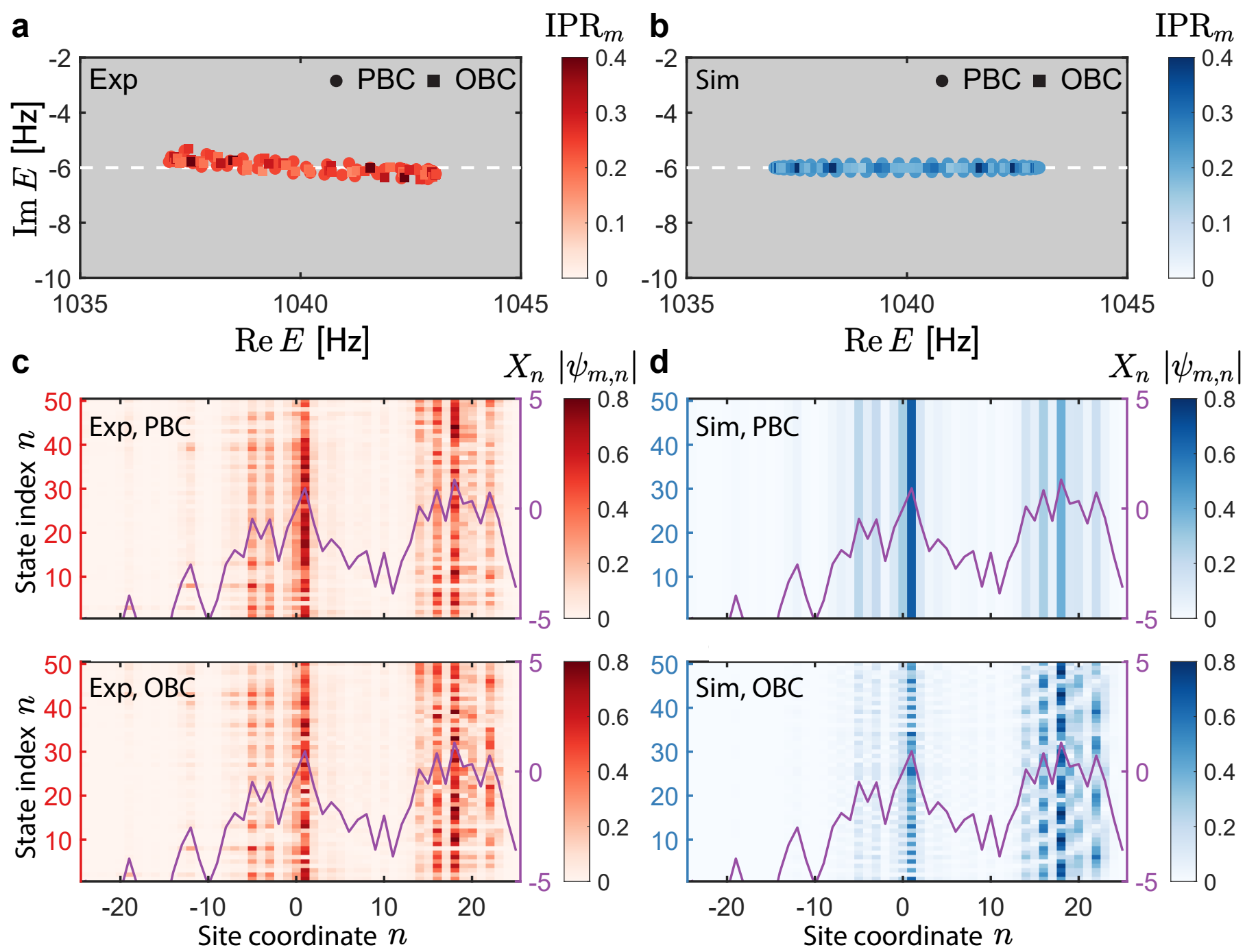

Figure S6: Spectral signatures of ENHSL, comparing experiments and tight-binding simulations for a chain of size $N = 50$ with $\omega_0 = 1040\,\mathrm{Hz} - 6i\,\mathrm{Hz}$, $J = 1.5\,\mathrm{Hz}$, and $\Delta h = 2\,\mathrm{Hz}$. a,b, Complex spectra for a representative realization under both PBC and OBC. Circles (squares) denote PBC (OBC) energies, and colors encode IPR_m. **c,d**, Spatial distributions of all eigenstates under PBC (top) and OBC (bottom) for experiment (c) and simulation (d). Heat maps show the eigenstate amplitudes $|\psi_{m,n}|$ as functions of eigenstate index m and site coordinate n. The overlaid purple curve (right axis) shows the random-walk landscape X_n.

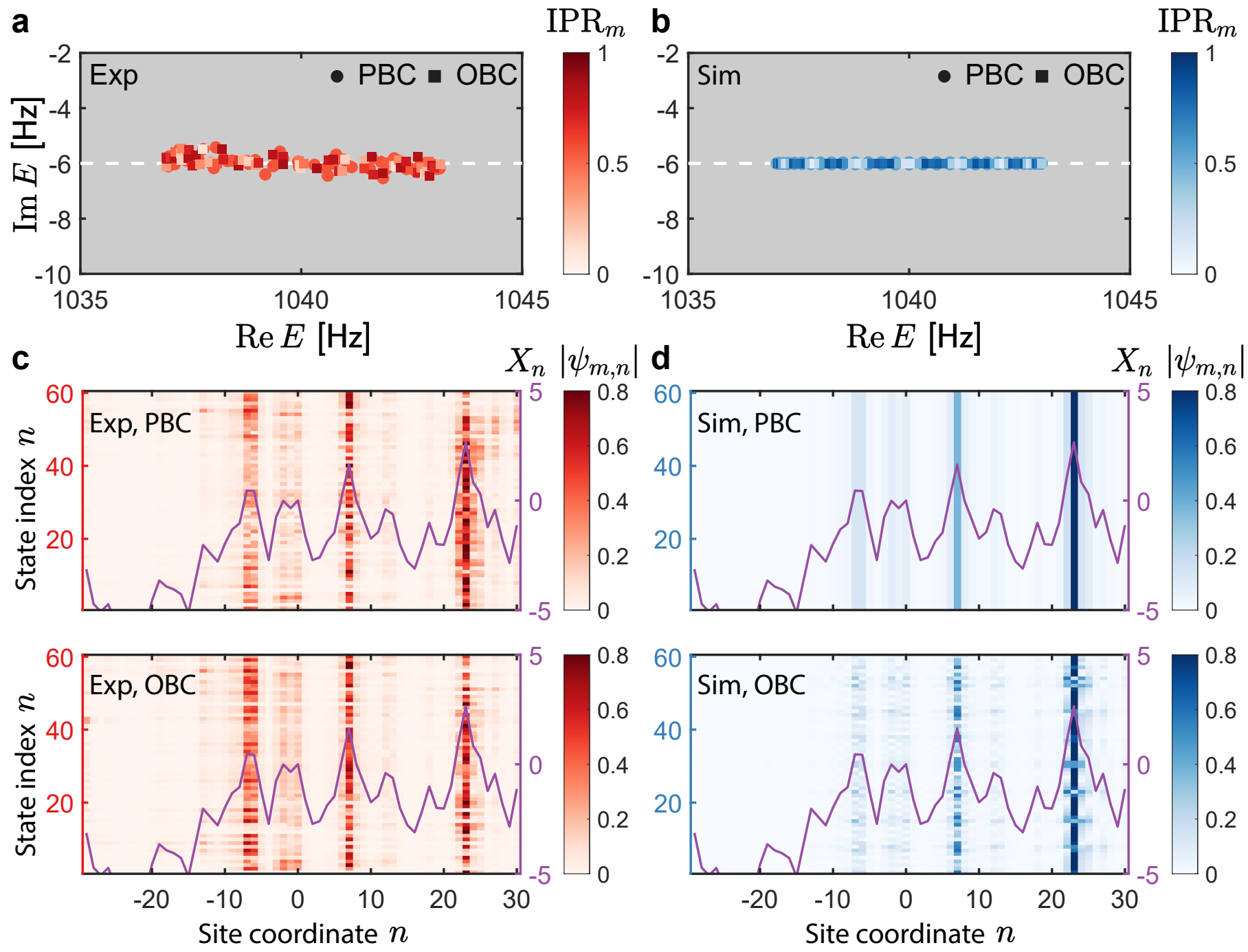

Figure S7: Spectral signatures of ENHSL, comparing experiments and tight-binding simulations for a chain of size $N = 60$ with $\omega_0 = 1040\,\mathrm{Hz} - 6i\,\mathrm{Hz}$, $J = 1.5\,\mathrm{Hz}$, and $\Delta h = 2\,\mathrm{Hz}$. a,b, Complex spectra for a representative realization under both PBC and OBC. Circles (squares) denote PBC (OBC) energies, and colors encode IPR_m. **c,d**, Spatial distributions of all eigenstates under PBC (top) and OBC (bottom) for experiment (c) and simulation (d). Heat maps show the eigenstate amplitudes $|\psi_{m,n}|$ as functions of eigenstate index m and site coordinate n. The overlaid purple curve (right axis) shows the random-walk landscape X_n.

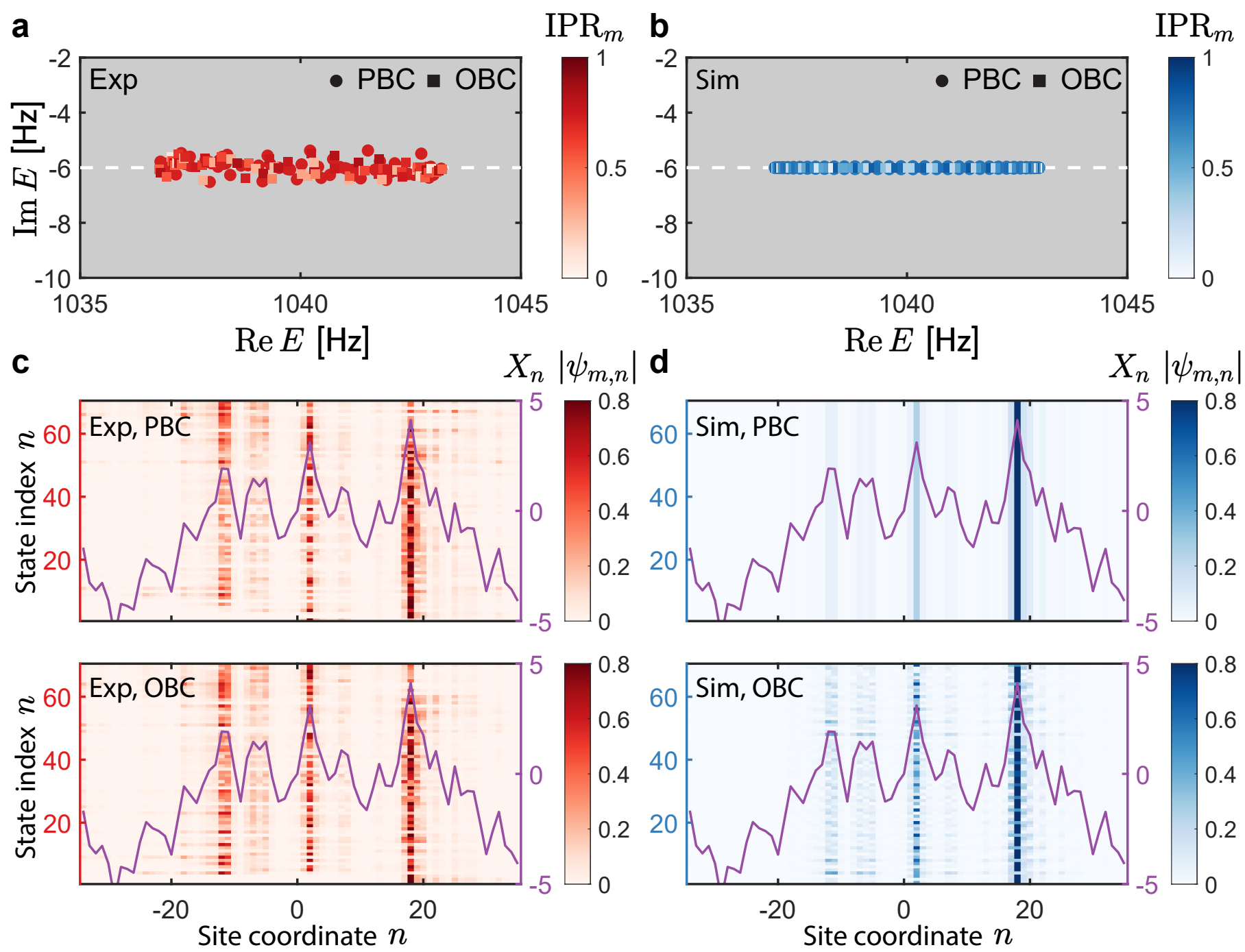

Figure S8: Spectral signatures of ENHSL, comparing experiments and tight-binding simulations for a chain of size $N = 70$ with $\omega_0 = 1040\,\mathrm{Hz} - 6i\,\mathrm{Hz}$, $J = 1.5\,\mathrm{Hz}$, and $\Delta h = 2\,\mathrm{Hz}$. a,b, Complex spectra for a representative realization under both PBC and OBC. Circles (squares) denote PBC (OBC) energies, and colors encode IPR_m. **c,d**, Spatial distributions of all eigenstates under PBC (top) and OBC (bottom) for experiment (c) and simulation (d). Heat maps show the eigenstate amplitudes $|\psi_{m,n}|$ as functions of eigenstate index m and site coordinate n. The overlaid purple curve (right axis) shows the random-walk landscape X_n.

Section S3. Extended experimental data for dynamical observations of ENHSL

Figure S9 presents additional representative experimental data for the dynamical observations of ENHSL, showing wave-packet propagation following excitation at the central site ($n = 0$) in a chain of length $N = 61$ under OBC for five different disorder realizations. It is clearly seen that the wave packet exhibits distinct propagation patterns depending on the specific disorder realization, reflecting the strong sample-to-sample fluctuations characteristic of ENHSL. In some realizations, the wave packet spreads rapidly and saturates slowly, while in others, the spreading is slower than the ballistic reference. These observations further corroborate the theoretical predictions regarding the rich dynamical behavior of ENHSL and highlight the significant influence of disorder realizations on wave-packet dynamics.

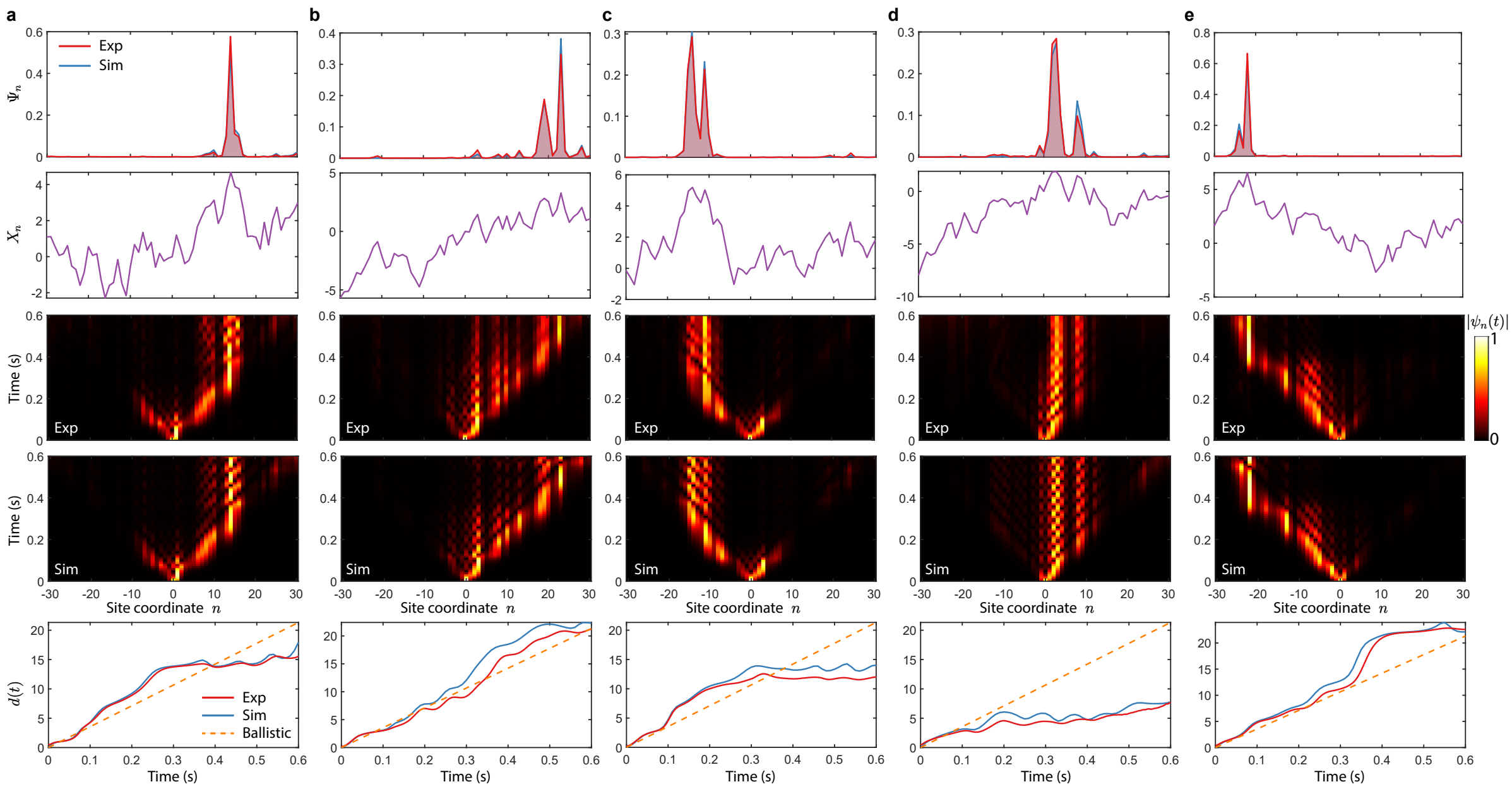

Figure S9: Experimental observation of wave-packet propagation following excitation at the central site ($n = 0$) in a chain of length $N = 61$ under OBC. The parameters set in experiments are $\omega_0 = 1040\,\text{Hz} - 1.75i\,\text{Hz}$, $J = 4\,\text{Hz}$, and $\Delta h = 1.6\,\text{Hz}$. **a–e**, Five representative disorder realizations of the ENHSL.

References

[1] Zhong, J.-X. *et al.* Experimentally probing non-Hermitian spectral transition and eigenstate skewness. *Phys. Rev. B* **112**, L220301 (2025).

[2] Zhong, J.-X., Roy, B. & Jing, Y. Observation of dislocation bound states and skin effects in non-Hermitian Chern insulators. *Adv. Mater.* **e15496**, e15496 (2026).
