# Supplementary material for: Observation of erratic non-Hermitian skin localization and transport
Source: Nat Commun. 2026 Aug 21;17:9979. doi: 10.1038/s41467-026-76908-3 (PMC13590536; doi:10.1038/s41467-026-76908-3)
Supplement: Supplementary file 2 [file 41467_2026_76908_MOESM2_ESM.pdf]

nature portfolio

Peer Review File

Observation of Erratic Non-Hermitian Skin Localization and Transport

Corresponding Author: Professor yun jing

This file contains all reviewer reports in order by version, followed by all author rebuttals in order by version.

Version 0:

Reviewer comments:

Reviewer #1

(Remarks to the Author)
This manuscript reports an experimental demonstration of a recently proposed localization phenomenon in non-Hermitian disordered systems, termed erratic non-Hermitian skin localization (ENHSL). This intriguing phenomenon is characterized by the coexistence of irregular eigenstate localization and dynamical ballistic transport. Such behavior is fundamentally distinct from both the Anderson localization and conventional NHSE, thereby enriching the landscape of localization physics in condensed matter systems. By constructing a flexibly tunable active acoustic lattice, the authors not only observe the disorder-dependent bulk localizations predicted in Ref. [40], but also develop and validate a complementary theoretical framework describing the associated dynamical ballistic transport. This combined investigation of both steady and dynamical aspects significantly deepens the physical understanding of ENHSL and broadens its conceptual scope. Overall, the study demonstrates both depth and breadth. As an experimental advance, it is timely and meaningful. The manuscript is logically organized and clearly written. The experimental implementation is carefully designed, and the measurements appear solid and convincing. In my view, the work meets the standard for publication in Nature Communications, provided that the following issues are reasonably addressed.

Main Comments
1. The authors use the disordered Hatano–Nelson model as an example to demonstrate ENHSL. However, we are not entirely clear about the universality of ENHSL, namely, in what class of systems ENHSL can generally arise. Beyond the requirement of global reciprocity, are there additional necessary conditions? For instance, can ENHSL occur in systems with purely unidirectional coupling or in systems with long-range hopping? We suggest that the authors provide a clearer and more systematic discussion on this point.

2. If we understand correctly, global reciprocity can ensure the absence of the conventional NHSE, even under OBCs. Then, if the system does not satisfy global reciprocity but still contains disordered nonreciprocal hopping, what typical phenomena would emerge? Can ENHSL and NHSE coexist in such a situation? If so, how should one distinguish between them? A clarification of this issue would help better position ENHSL relative to the conventional NHSE.

3. In Fig. 1, the authors should specify whether the model is considered under OBCs or PBCs. If PBCs are assumed, the schematic in Fig. 1a would be better illustrated in a ring geometry. In addition, the authors state that the spectrum is insensitive to boundary conditions (OBCs or PBCs). It would be helpful if they could further explain the weak sensitivity of the eigenstates to boundary conditions and provide the underlying physical reason.

4. In Fig. 3b, both the OBC and PBC spectra of the model are real. It is easy to understand why the OBC spectrum is real, since the corresponding nonreciprocal Hamiltonian is related to a reciprocal one through a similarity transformation. However, the fact that the PBC spectrum also remains real is not immediately obvious. Could the authors explain or demonstrate why this is the case?

5. In Fig. 4a and Fig. 4c, the two ENHSL realizations exhibit similar spatial profiles of the eigenstate intensity. However, the corresponding spatiotemporal evolutions show a clear difference: in realization 1, the acoustic pressure eventually localizes at the dominant peak, whereas in realization 3, it appears more extended. Could the authors provide a more specific explanation for this difference?

6. In fact, the Green's-function-based spectroscopy technique for reconstructing the full complex spectrum and eigenstates is not limited to Ref. [46]. Similar approaches have been employed in circuit systems (e.g., Nat. Phys. 16, 747 (2020)) and in other acoustic works (e.g., Phys. Rev. Lett. 134, 126603 (2025)). Moreover, previous studies on active acoustic lattices have demonstrated simultaneous control of nonreciprocal coupling amplitude and phase (e.g., Phys. Rev. Lett. 017201 (2023)), which is more directly related to the experimental techniques used in the present work. We suggest that the authors handle the literature citations in a more balanced and appropriate manner.

Minor Comments
1. In the last row panels of Fig. 4, the correspondence between experiment and simulation in the legend is inconsistent with that described in the caption.

2. Both the Bessel function and the hopping amplitude are denoted as Jn, which may cause confusion.

3. In the caption of Fig. 3, both J and Δh are given in units of Hz. If J already carries units of Hz, then h and the corresponding Δh should presumably be dimensionless. Please clarify the units for consistency.

Reviewer #2

(Remarks to the Author)
I co-reviewed this manuscript with one of the reviewers who provided the listed reports. This is part of the Nature Communications initiative to facilitate training in peer review and to provide appropriate recognition for Early Career Researchers who co-review manuscripts.

Reviewer #3

(Remarks to the Author)
In this manuscript, the authors present an experimental realization of erratic non-Hermitian skin localization (ENHSL) in a one-dimensional lattice. They implement a platform where both spectral properties and wave-packet dynamics are measured, showing that eigenstates exhibit irregular bulk localization while the average transport remains ballistic. The experimental results are compared with theoretical predictions and show good agreement.

The theoretical concept of ENHSL has been recently proposed and analyzed, and this work provides an experimental demonstration of the phenomenon. The measurement protocol, combining spectral reconstruction with dynamical observation, is carefully implemented. However, the manuscript does not reveal fundamentally new insights beyond the original theoretical predictions. Consequently, the study is primarily an experimental validation of an existing theoretical prediction. The authors highlight the coexistence of ballistic transport and strong spectral localization, which is actually a standard behavior in non-Hermitian systems with the skin effect. That is, non-reciprocity drives an initial state to the positions where skin modes are localized: boundaries for conventional NHSE, and those peaks inside the bulk for ENHSL.

Overall, the work represents a solid experimental study, but its conceptual novelty is moderate. While the approach and measurements are of interest to researchers studying non-Hermitian systems, the scientific advance may be considered incremental for more broader audience. As such, I cannot recommend publication in Nature Communications.

An addition question:
The terminology "spectral localization" is confusing. I see clear spatial localization in the figures; but the spectra in Fig. 1 (schematic) and 3 (exp. and sim.) do not show anything special: they just form a continuum band on the complex energy plane. So what does "spectral" in this phrase imply?

Reviewer #4

(Remarks to the Author)
The manuscript investigates a non-Hermitian chain with disordered nonreciprocal hopping. For comparison, the authors analyze the eigenstates and dynamical behavior relative to classical Anderson localization. They theoretically identify a distinct localization phenomenon—erratic non-Hermitian skin localization (ENHSL)—and corroborate it experimentally using an acoustic platform. However, despite the novelty of ENHSL, the underlying mechanism is not explained with sufficient clarity. The experimental measurement of Green's function matrix is also well developed in their previously published paper(Ref. 46) and does not, by itself, constitute a substantive experimental advance. For these reasons, I do not consider the work suitable for publication in Nature Communications, owing to limited experimental novelty and insufficient theoretical development. The following issues should be addressed before the manuscript is submitted to another journal.

1. What is the underlying mechanism of ENHSL? Specifically, what's the meaning of "the spectrum remains approximately real" (as noted in the caption of Fig. 1), and is the spectrum dependent on system size?
2. In Fig. 1(c,e), the eigenstates display two dominant peaks localized at the left and right edges, and the dynamical responses show bidirectional wave-packet propagation, with the right peak being slightly larger. What accounts for this asymmetry? Were the results averaged over multiple disorder realizations?
3. In Fig. 4, the spreading distance d(t) for case (c) shows a markedly different trend from cases (a) and (b). Does this imply that ENHSL is highly sensitive to sample configuration?

Version 1:

Reviewer comments:

Reviewer #1

(Remarks to the Author)
The authors have adequately addressed my concerns and suggestions, and I thus recommend publication.

Reviewer #2

(Remarks to the Author)
I co-reviewed this manuscript with one of the reviewers who provided the listed reports. This is part of the Nature Communications initiative to facilitate training in peer review and to provide appropriate recognition for Early Career Researchers who co-review manuscripts.

Reviewer #3

(Remarks to the Author)
In response to my previous report, the authors highlight two contributions of the manuscript: "(i) the first experimental observation of a new form of localization (ENHSL) and (ii) the discovery of its unexpected ballistic transport regime in the globally reciprocal case".

To me, point (i) by itself is not sufficient for publication in Nature Communications. In the response, the authors also emphasize that "the present work is not a simple experimental verification of the recently proposed ENHSL mechanism". Therefore, the issue at hand is the level of novelty of point (ii).

In my previous report, I said that it is "a standard behavior in non-Hermitian systems with the skin effect", driven by the non-reciprocal pumping related to NHSE or NHESL. The authors refutes this by emphasizing that the ballistic transport unexpectedly exist in the globally reciprocal regime, which is "a fully novel and intrinsically nontrivial dynamical phenomenon".

However, after digesting the details, I find this phenomenon not as unexpected as the authors suggest. First, globally reciprocal refers to zero average nonreciprocity -- which means that the system is still locally nonreciprocal. In fact, this is why I think the dynamics is standard in the first place: the disordered nonreciprocity gives local peaks inside the bulk, where the wave packet flows to. Its presence under global reciprocity may sounds uncommon: at a single experiment one may observe leftward or rightward ballistic transport, but the ensemble-averaged behaviors shall reflect the globally reciprocal nature. However, the dynamics is quantified by the second moment, which is proportional to the absolute value of the displacement from n=0. So leftward and rightward ballistic transports, which can both be understood from the "funneling" picture, are unified to give a linear dependence of the second moment on time.

The authors also stress that the "funneling" picture is limited, as it predicts "final dynamical localization (attraction) around some erratic site in the bulk, not ballistic motion". However, this picture actually does predict ballistic motion before the wave packet reaching the "global extremum of the random-walk landscape". As the authors point out in their response, a global maximum "can occur arbitrarily far from the initial position, and its distance fluctuates strongly between disorder realizations". That is, different cases in an ensemble have different distances between global maximum and the initial position, so they requires different time for the ballistic transport to stop at the maximum. After averaging, the slope of the displacement-versus-time curve is expected to decrease over time, since some cases reach the maximum earlier while others do so later. This is actually what Fig. 5 in the manuscript shows, where the blue and red curves slightly drops over time.

For the above reasons, I am not convinced that the finding "goes significantly beyond all previously known theoretical predictions", as the authors claim. Rather, it seems to be a comprehension of some known phenomena with a bit different theoretical tools. Therefore I cannot recommend publication in Nature Communications.

Reviewer #4

(Remarks to the Author)
I have carefully reviewed the authors' response and the revised manuscript. The previously raised concerns have been satisfactorily addressed, and I have no further comments.

Open Access This Peer Review File is licensed under a Creative Commons Attribution 4.0 International License, which permits use, sharing, adaptation, distribution and reproduction in any medium or format, as long as you give appropriate credit to the original author(s) and the source, provide a link to the Creative Commons license, and indicate if changes were made.
In cases where reviewers are anonymous, credit should be given to 'Anonymous Referee' and the source.
The images or other third party material in this Peer Review File are included in the article's Creative Commons license, unless indicated otherwise in a credit line to the material. If material is not included in the article's Creative Commons license and your intended use is not permitted by statutory regulation or exceeds the permitted use, you will need to obtain permission directly from the copyright holder.
To view a copy of this license, visit https://creativecommons.org/licenses/by/4.0/

Responses to Reviewers' Comments

Re: Manuscript ID NCOMMS-26-008273-T

"Observation of Erratic Non-Hermitian Skin Localization and Transport"

We have carefully revised the manuscript to address all the reviewers' comments. A summary of the key changes is tabulated below.

Table R1. List of major changes in the revised manuscript. Rev$i$-Cj denotes Comment j from Reviewer i; Rev$i$-G denotes the general comment from Reviewer i. All changes are marked in red in the revised manuscript.

Source	Topic of concern	Revision & Location
Rev1-C1, Rev3-G, Rev4-G	Universality and theoretical scope of ENHSL	**Added a new Methods subsection, "General theoretical framework of the ENHSL",** showing that ENHSL is not restricted to the disordered Hatano-Nelson model, but arises generally from imaginary-gauge deformations of Hermitian parent lattices under global reciprocity. This expands the theoretical foundation and clarifies the general criteria for ENHSL.
Rev1-C2, Rev4-C1	Relation between ENHSL, global reciprocity, and the conventional NHSE	**Revised the "Model and conceptual overview" section** to introduce the mean imaginary gauge field $\overline{h} = (1/N)\sum_n h_n$. We now explain that $\overline{h} \neq 0$ gives the conventional NHSE, whereas $\overline{h} = 0$ is the globally reciprocal transition point where the conventional NHSE disappears and ENHSL emerges.
Rev1-C2, Rev4-C1	Mechanism of ENHSL and subexponential bulk localization	**Added a detailed explanation of the random-walk mechanism.** In the globally reciprocal case, $X_n = \sum_\ell h_\ell$ is an unbiased random walk with vanishing drift/Lyapunov exponent, preventing ordinary exponential edge accumulation. Large excursions of X_n instead generate realization-dependent, subexponential bulk localization peaks.

Continued on next page

Table R1 – continued from previous page

Source	Topic of concern	Revision & Location
Rev1-C3, Rev1-C4, Rev4-C1	Boundary-condition dependence and real spectrum under PBC/OBC	**Clarified why PBC and OBC spectra are real and asymptotically coincident under global reciprocity.** The new Methods discussion shows that for $\overline{h} \neq 0$, the PBC spectrum follows the complex deformation $k \to k - i\overline{h}$, leading to nonzero winding and edge accumulation; for $\overline{h} = 0$, the winding vanishes and the PBC/OBC spectra become real and identical in the thermodynamic limit.
Rev1-C3, Rev4-C2	Interpretation of Fig. 1 and schematic boundary conditions	**Revised Fig. 1 caption** to state that panel (a) represents the OBC geometry used in the experiment, and that panels (c) and (e) are schematic illustrations of a single representative disorder realization rather than disorder-averaged results. We also clarified that the peak asymmetry in Fig. 1 reflects realization-specific fluctuations of X_n, not boundary skin accumulation or a net drift.
Rev3-C1	Meaning of "spectral localization"	**Clarified the terminology in the Introduction.** We now explicitly state that "spectral localization" refers to spatial localization of all Hamiltonian eigenstates, in contrast to dynamical localization or transport suppression.
Rev2-G, Rev3-G, Rev4-G	Novelty beyond the previous ENHSL theory	**Revised the Introduction and theory sections** to emphasize that the present work goes beyond the original spectral theory of ENHSL by developing and experimentally verifying a new dynamical theory. In particular, we highlight ballistic ensemble transport, dominant-site selection, and Lévy-arcsine statistics as central new results not predicted in the previous work.
Rev3-G, Rev4-G	Dynamical mechanism beyond a simple "funnelling" picture	**Expanded the main-text dynamics theory.** We now show that the non-unitary gauge transformation maps the dynamics to a Hermitian ballistic propagator modulated by a random-walk envelope, and explain why the dynamics is controlled by extreme-value statistics within the finite-time light cone rather than by attraction to a fixed global maximum.

Continued on next page

Table R1 – continued from previous page

Source	Topic of concern	Revision & Location
Rev3-G, Rev4-G	Experimental advance beyond Green's-function spectroscopy alone	**Clarified the unified spectral-dynamical protocol.** We emphasize that the experimental advance is not only Green's-function-based spectral reconstruction, but the ability to combine full complex-spectrum/eigenstate measurements with time-resolved wave-packet dynamics on the same acoustic platform and for the same disorder realization.
Rev1-C5, Rev4-C3	Realization-dependent dynamics and different $d(t)$ trends in Fig. 4	**Added clarification in the "Experimental observation of single-realization dynamics" section.** We explain that $d(t)$ can differ substantially between individual ENHSL realizations because it is sensitive to weak satellite structures and transient weight at larger $\|n\|$. The more extended behaviour in Fig. 4c is therefore a sample-specific ENHSL feature, not an experimental artefact.
Rev1-C6	Missing related references	**Added the suggested references** on related Green's-function reconstruction and active acoustic non-Hermitian platforms.
Rev1-C7	Figure legend, notation, and units	**Corrected several presentation and notation issues.** We revised the Fig. 4 caption to match the plotted legend, changed the Bessel-function notation to $\mathcal{J}_n$ to avoid confusion with hopping amplitudes $J_n^{\mathrm{L/R}}$, and clarified that Δh is dimensionless.

1 Comments from Reviewer 1

1.1 General comment

This manuscript reports an experimental demonstration of a recently proposed localization phenomenon in non-Hermitian disordered systems, termed erratic non-Hermitian skin localization (ENHSL). This intriguing phenomenon is characterized by the coexistence of irregular eigenstate localization and dynamical ballistic transport. Such behavior is fundamentally distinct from both the Anderson localization and conventional NHSE, thereby enriching the landscape of localization physics in condensed matter systems. By constructing a flexibly tunable active acoustic lattice, the authors not only observe the disorder-dependent bulk localizations predicted in Ref. [40], but also develop and validate a complementary theoretical framework describing the associated dynamical ballistic transport. This combined investigation of both steady and dynamical aspects significantly deepens the physical understanding of ENHSL and broadens its conceptual scope. Overall, the study demonstrates both depth and breadth. As an experimental advance, it is timely and meaningful. The manuscript is logically organized and clearly written. The experimental implementation is carefully designed, and the measurements appear solid and convincing. In my view, the work meets the standard for publication in Nature Communications, provided that the following issues are reasonably addressed.

Responses

We sincerely thank the referee for the positive and encouraging assessment of our work. We are grateful that the referee recognizes (i) the conceptual significance of erratic non-Hermitian skin localization (ENHSL), (ii) the clear distinction between ENHSL, Anderson localization, and the conventional non-Hermitian skin effect (NHSE), and (iii) the value of combining steady-state eigenstate measurements with dynamical transport experiments. We also appreciate the referee's acknowledgement of the careful design of our active acoustic lattice and the clarity of the manuscript.

In the revised version, we have addressed all issues raised by the referee. We have expanded the theoretical exposition of ENHSL, clarified the disorder-dependent localization characteristics, strengthened the presentation and justification of the dynamical ballistic transport framework, and made several textual improvements to enhance readability. We hope that these revisions further improve the manuscript and fully meet the standard for publication in *Nature Communications*, as suggested by the referee.

1.2 Main comment 1

> The authors use the disordered Hatano-Nelson model as an example to demonstrate ENHSL. However, we are not entirely clear about the universality of ENHSL, namely, in what class of systems ENHSL can generally arise. Beyond the requirement of global reciprocity, are there additional necessary conditions? For instance, can ENHSL occur in systems with purely unidirectional coupling or in systems with long-range hopping? We suggest that the authors provide a clearer and more systematic discussion on this point.

Responses

We thank the referee for raising this important question regarding the universality and general conditions for the emergence of ENHSL. In the revised manuscript, we have expanded the theoretical part on the erratic ENHSL, adding a new subsection in the Methods to give a clearer and more systematic account of the general criteria and universality for ENHSL.

We summarize and clarify the key points as follows:

1. *Global reciprocity is the only essential structural requirement.*

ENHSL relies on the existence of a *globally reciprocal* (i.e., net-reciprocal) non-Hermitian lattice, such that the overall system does not possess a preferred direction for bulk transport. This condition suppresses the conventional NHSE and allows disorder-induced fluctuations in the effective hopping asymmetry to control localization. Formally, global reciprocity is signaled by a sign change in a topological winding number [1,2], which determines the collapse direction of skin modes: the orientation reversal of the winding leads to a reversal of collapse direction of skin modes. It is precisely at the topological phase transition point that the ENHSL sets in.

2. *Purely unidirectional (fully chiral) models.*

The limit of a purely unidirectional (fully chiral) chain is a rather special and highly-degenerate case of the random Hatano-Nelson model considered in our work, where adjacent sites in the lattice are unidirectionally coupled with a rate T stochastically to the left or to the right. This fully chiral model, with chirality randomly switching from left to right along the lattice, can be obtained from our model considering a Bernoulli distribution for h_n [h_n can take with the same probability the two values $\pm h$] in the double limit $h \to \infty$ and $J \to 0$, with $J \exp(h) = T$ finite and non-zero. In this singular limit the eigenvectors become defective (we have exceptional points) and are compact, localized at the sites where chirality flips. The energy spectrum collapses to the single zero energy point, and ballistic transport is prevented (according to our analysis in the limit $J \to 0$).

3. *Long-range hopping is compatible with ENHSL.*

The ENHSL can occur in non-Hermitian systems with long-range hopping, provided that the summed hopping amplitudes satisfy global reciprocity. We have added a brief theoretical justification

4. *Universality.*

As we demonstrate in the revised manuscript, ENHSL generically arise in a broad class of globally reciprocal non-Hermitian lattices at a topological phase transition point, irrespective of range of hopping, number of bands, or specific realization- because it originates from disorder-induced spatial fluctuations of the superimposed imaginary gauge field that dominate over a vanishing net drift. It can also arise in higher-dimensional (2D or 3D) lattice models, such as the 2D stochastic Hatano-Nelson model with nearest-neighbor or long-range hopping.

These clarifications have been added to the revised manuscript and elaborated in Methods section.

Changes

- A new subsection entitled "General theoretical framework of the ENHSL" has been added in the Methods section, providing a clearer and more systematic discussion of the general criteria and universality for ENHSL.

1.3 Main comment 2

> If we understand correctly, global reciprocity can ensure the absence of the conventional NHSE, even under OBCs. Then, if the system does not satisfy global reciprocity but still contains disordered nonreciprocal hopping, what typical phenomena would emerge? Can ENHSL and NHSE coexist in such a situation? If so, how should one distinguish between them? A clarification of this issue would help better position ENHSL relative to the conventional NHSE.

Responses

We thank the referee for raising this important issue, which we have clarified in the revised manuscript. In a lattice with disordered non-reciprocal hopping and global non-reciprocity, all eigenstates become exponentially localized at one lattice edge, with a non-vanishing Lyapunov exponent, indicating edge exponential localization, a hallmark of the NHSE. While in a clean non-reciprocal lattice the eigenvectors are purely exponential, in a disordered globally non-reciprocal lattice the eigenvector profile is modulated by the envelope term

$$\exp(X_n) = \exp\left(\sum_{l=0}^{n} h_l\right) = \exp\left(\overline{h}n\right)\,\exp\left(\sum_{l=0}^{n} \tilde{h}_l\right),$$

where $\overline{h} = (1/N)\sum_{l=1}^{N} h_l$ is the mean non-reciprocity, and $\tilde{h}_n = h_n - \overline{h}$ is a zero-mean random field. The resulting eigenvector profile is a generally non-monotonous exponential, i.e., an exponential term $\exp\left(\overline{h}n\right)$ that concentrates the excitation toward the lattice edge, modulated by an erratic factor $\exp\left(\sum_{l=0}^{n} \tilde{h}_l\right)$ with local maxima and minima. Conversely, in the globally reciprocal case, the localization is *sub-exponential*, as indicated by the vanishing Lyapunov exponent. It is determined solely by the extreme-value statistics of the symmetric random walk $X_n = \sum_{l=0}^{n} h_l$ and occurs erratically in the bulk, rather than at the lattice edge.

As for the dynamical aspect: under global non-reciprocity, the wave packet undergoes a uni-directional (biased) drift—either to the left or to the right, depending on the sign of $\overline{h}$—characteersitic of systems exhibiting the NHSE In contrast, in the globally-reciprocal case the wave packet center of mass remains on average at rest. This provides the clearest dynamical signature distinguishing the two regimes.

This point has been clarified in the revised manuscript.

Changes

- Lines 88–99 in the revised manuscript. We clarified in the "Model and conceptual overview" section that the mean imaginary gauge field $\overline{h} = (1/N)\sum_n h_n$ quantifies global non-reciprocity. We now explicitly state that $\overline{h} \neq 0$ gives the conventional NHSE with biased bulk transport, whereas $\overline{h} = 0$ is the globally reciprocal transition point where the NHSE disappears and ENHSL emerges.
- Lines 103–123 in the revised manuscript. We added a direct comparison between the globally non-reciprocal and globally reciprocal cases. For $\overline{h} \neq 0$, the random-walk landscape is biased and the system shows the usual NHSE signatures: boundary-sensitive spectra, non-vanishing winding number, exponential edge localization under OBC, and biased transport. In contrast, for $\overline{h} = 0$, the random walk is unbiased, the Lyapunov exponent vanishes, and localization becomes subexponential and erratic in the bulk, driven by large excursions of X_n.
- We expanded the Methods with a new subsection, "General theoretical framework of the ENHSL", which shows how imaginary-gauge deformations of a Hermitian parent lattice generate disordered non-Hermitian companion models. This framework explicitly explains why nonzero $\overline{h}$ yields the conventional NHSE, whereas global reciprocity $\overline{h} = 0$ removes the macroscopic boundary drift and produces bulk ENHSL peaks.
- We revised Fig. 1 and its caption to make the boundary-condition distinction clearer.

1.4 Main comment 3

> In Fig. 1, the authors should specify whether the model is considered under OBCs or PBCs. If PBCs are assumed, the schematic in Fig. 1a would be better illustrated in a ring geometry. In addition, the authors state that the spectrum is insensitive to boundary conditions (OBCs or PBCs). It would be helpful if they could further explain the weak sensitivity of the eigenstates to boundary conditions and provide the underlying physical reason.

Responses

We thank the referee for this comment. In the revised manuscript we have clarified the role of boundary conditions in Fig. 1. Figure 1(a) illustrates the physical structure of the system as a linear chain with open boundary conditions (OBC). The schematic spectrum in Fig. 1(c), on the other hand, refers to the energy spectrum of the corresponding globally reciprocal non-Hermitian lattice and can represent either periodic (PBC) or open (OBC) boundary conditions. In the globally reciprocal case, characterized by a vanishing mean imaginary gauge field $\overline{h} = 0$, the system does not exhibit the NHSE. As demonstrated in the new subsection added to the Methods, the energy spectrum and eigenvectors become insensitive to the choice of boundary conditions in the thermodynamic limit $N \to \infty$. This boundary-condition insensitivity is a direct consequence of the absence of any extensive nonreciprocal drift: since the net imaginary gauge bias vanishes, there is no mechanism that can accumulate the eigenstates at a boundary, and hence PBC and OBC spectra converge to the same real set $\sigma(H)$ as N grows.

Only when $\overline{h} \neq 0$, i.e., when the system is globally non-reciprocal, does the conventional NHSE arise. In that case, spectral and eigenstate properties under OBC and PBC differ markedly, and one must clearly specify which boundary condition is used when plotting spectra or eigenvectors.

For finite lattice size N, even in the globally reciprocal case, the PBC spectrum can exhibit weak finite-size deviations from the real OBC spectrum, which are slightly disorder-dependent. These deviations vanish as N increases, as one can readily verified by numerical simulations. This behavior reflects only sub-extensive corrections, not a genuine skin effect. This clarification has now been incorporated in the caption and description of Fig. 1.

Changes

- A new subsection entitled "General theoretical framework of the ENHSL" has been added in the Methods section, providing a clearer and more systematic discussion of the insensitivity of the spectrum and eigenstates to boundary conditions in the globally reciprocal case.
- We revised Fig. 1 and its caption to make the boundary-condition distinction clearer.

1.5 Main comment 4

> In Fig. 3b, both the OBC and PBC spectra of the model are real. It is easy to understand why the OBC spectrum is real, since the corresponding nonreciprocal Hamiltonian is related to a reciprocal one through a similarity transformation. However, the fact that the PBC spectrum also remains real is not immediately obvious. Could the authors explain or demonstrate why this is the case?

Responses

We thank the Referee for this remark and suggestion. In the revised mansucript, first section of Methods, we have now explicitly demonstrated that the PBC energy spectrum remains real even under PBC whenever the system is globally reciprocal.

Changes

- A new subsection entitled "General theoretical framework of the ENHSL" has been added in the Methods section, providing a clearer and more systematic discussion of the insensitivity of the spectrum and eigenstates to boundary conditions in the globally reciprocal case.

1.6 Main comment 5

> In Fig. 4a and Fig. 4c, the two ENHSL realizations exhibit similar spatial profiles of the eigenstate intensity. However, the corresponding spatiotemporal evolutions show a clear difference: in realization 1, the acoustic pressure eventually localizes at the dominant peak, whereas in realization 3, it appears more extended. Could the authors provide a more specific explanation for this difference?

Responses

We thank the referee for this thoughtful question. The observed difference between the spatiotemporal evolutions in Fig. 4a and Fig. 4c arises from the intrinsically disorder-dependent nature of ENHSL. While the two realizations shown in Fig. 4a and Fig. 4c display broadly similar eigenstate intensity profiles, the detailed structure of their eigenmodes is nevertheless sensitive to the specific realization of the fluctuating imaginary gauge fields h_n. This sensitivity is precisely why we refer to the phenomenon as "erratic": the eigenstates and the corresponding dynamics can vary appreciably from one disorder realization to another.

In the realization displayed in Fig. 4c (realization 3), the reconstructed eigenstates exhibit weak but non-negligible lateral satellite peaks on the right side of the lattice, which are absent in the case of Fig. 4a (realization 1). These satellite peaks arise from the fluctuations of the zero-mean

random field $\tilde{h}_n$ and provide additional probability weight toward the right side. As a result, during the time evolution the excitation in Fig. 4c spreads slightly further to the right before being dominated by the main peak, giving the impression of a more extended profile compared to Fig. 4a, where no such satellite structures are present.

We emphasize that despite these realization-dependent differences, the averaged dynamics over many disorder configurations remains ballistic, as discussed in the manuscript. This is one of the most striking and conceptually novel aspects of ENHSL: while each realization exhibits highly irregular, sample-specific localization features, the ensemble-averaged transport is cleanly ballistic.

Changes

- Lines 294–300 in the revised manuscript. We have added a clarification in the "Experimental observation of single-realization dynamics" section explaining that the more extended appearance of Fig. 4c arises from weak satellite structures in the reconstructed eigenstate profile, which provide additional transient weight away from the dominant peak.

1.7 Main comment 6

> In fact, the Green's-function-based spectroscopy technique for reconstructing the full complex spectrum and eigenstates is not limited to Ref. [46]. Similar approaches have been employed in circuit systems (e.g., Nat. Phys. 16, 747 (2020)) and in other acoustic works (e.g., Phys. Rev. Lett. 134, 126603 (2025)). Moreover, previous studies on active acoustic lattices have demonstrated simultaneous control of nonreciprocal coupling amplitude and phase (e.g., Phys. Rev. Lett. 017201 (2023)), which is more directly related to the experimental techniques used in the present work. We suggest that the authors handle the literature citations in a more balanced and appropriate manner.

Responses

We thank the referee for pointing out the relevant literature and for the suggestion to provide a more balanced citation of previous works. In the revised manuscript, we have expanded the discussion of the Green's-function-based spectroscopy technique to include references to the cited works in circuit systems and acoustic lattices. We have also clarified how our implementation relates to and builds upon these previous studies, while highlighting the unique aspects of our approach in the context of ENHSL.

Changes

- Lines 67–72 in the revised manuscript. We revised the Introduction to provide a more balanced citation of previous Green's-function-based and full-response-matrix reconstruction approaches in non-Hermitian systems. We revised the description of the acoustic platform to acknowledge previous progress in active acoustic lattices, including the simultaneous control of nonreciprocal coupling amplitudes and phases.
- Lines 67–72, 83–85 in the revised manuscript. We clarified that Green's-function-based spectroscopy itself is not the sole experimental advance of the present work. Rather, our key experimental contribution is to combine full complex-spectrum/eigenstate reconstruction with time-resolved wave-packet measurements on the same active acoustic platform and for the same programmed disorder realization.

1.8 Minor comments

> 1. In the last row panels of Fig. 4, the correspondence between experiment and simulation in the legend is inconsistent with that described in the caption.
>
> 2. Both the Bessel function and the hopping amplitude are denoted as J_n, which may cause confusion.
>
> 3. In the caption of Fig. 3, both J and Δh are given in units of Hz. If J already carries units of Hz, then h and the corresponding Δh should presumably be dimensionless. Please clarify the units for consistency.

Responses

We thank the referee for these helpful minor comments. We have carefully revised the manuscript to address each of these points. Specifically, we have corrected the legend in Fig. 4 to ensure consistency with the caption, clarified the notation to distinguish between the Bessel function and the hopping amplitude, and confirmed that the unit for Δh is indeed dimensionless.

Changes

- The caption of Fig. 4 has been revised to ensure consistency with the legend.
- The notation for the Bessel function has been revised to $\mathcal{J}_n$ to distinguish it from the hopping amplitude $J_n^{\mathrm{L,R}}$.
- The unit for Δh has been set to dimensionless.

2 Comments from Reviewer 2

2.1 General comments

> I co-reviewed this manuscript with one of the reviewers who provided the listed reports. This is part of the Nature Communications initiative to facilitate training in peer review and to provide appropriate recognition for Early Career Researchers who co-review manuscripts.

Responses

Thank you for co-reviewing this manuscript. We have carefully considered the reports provided by the co-reviewer and have addressed all comments and suggestions in our revised manuscript. We believe that the revisions have strengthened the clarity, rigor, and overall quality of our work, and we hope that it now meets the standards for publication in Nature Communications.

3 Comments from Reviewer 3

3.1 General comments

> In this manuscript, the authors present an experimental realization of erratic non-Hermitian skin localization (ENHSL) in a one-dimensional lattice. They implement a platform where both spectral properties and wave-packet dynamics are measured, showing that eigenstates exhibit irregular bulk localization while the average transport remains ballistic. The experimental results are compared with theoretical predictions and show good agreement.
>
> The theoretical concept of ENHSL has been recently proposed and analyzed, and this work provides an experimental demonstration of the phenomenon. The measurement protocol, combining spectral reconstruction with dynamical observation, is carefully implemented. However, the manuscript does not reveal fundamentally new insights beyond the original theoretical predictions. Consequently, the study is primarily an experimental validation of an existing theoretical prediction. The authors highlight the coexistence of ballistic transport and strong spectral localization, which is actually a standard behavior in non-Hermitian systems with the skin effect. That is, non-reciprocity drives an initial state to the positions where skin modes are localized: boundaries for conventional NHSE, and those peaks inside the bulk for ENHSL.
>
> Overall, the work represents a solid experimental study, but its conceptual novelty is moderate. While the approach and measurements are of interest to researchers studying non-Hermitian systems, the scientific advance may be considered incremental for more broader audience. As such, I cannot recommend publication in Nature Communications.

Responses

We thank the referee for the careful and critical evaluation of our paper, and for considering our work a solid experimental study . We respectfully disagree with the assessment that the present manuscript constitutes a merely incremental experimental validation, and we clarify below why we believe the results are timely, conceptually significant, novel, and of broad relevance.

First, we emphasize that the present work is not a simple experimental verification of the recently proposed ENHSL mechanism predicted in a recent PRL paper by one of the present authors. While the experimental realization is indeed important in its own right, given that ENSHL is a genuinely new type of localization occurring at a topological phase transition point in globally-reciprocal non-Hermitian systems, the main impact of the present study lies in the discovery and direct observation of a *dynamical transport regime* that was not predicted or analyzed in the original theoretical work. Even as a standalone experimental demonstration of a previously unobserved and genuinely new localization phenomenon, we believe the results would already be of strong interest to a broad readership in non-Hermitian and wave physics due to the absence of any prior experimental observation of ENHSL.

Second, we stress that the topic is currently highly active and is rapidly evolving. Since the submission of our manuscript, several related works have appeared on arXiv exploring extensions and experimental realizations of erratic skin localization phenomena, including a recent photonic implementation (`https://arxiv.org/abs/2604.00088`) and a phononic implementation (`https://arxiv.org/abs/2604.23713`). This rapid emergence of independent activity highlights the timeliness and broad interest in the underlying, but not fully understood, physical mechanism.

Third, and most importantly, the present work uncovers a fully novel and intrinsically nontrivial dynamical phenomenon in the globally reciprocal regime—one that goes significantly beyond all previously known theoretical predictions. We demonstrate, both theoretically and experimentally, that exactly at the topological phase–transition point of global reciprocity—where the conventional NHSE is absent—one finds the unexpected coexistence of *ballistic* transport with spectrally localized eigenstates. This phenomenon has not been predicted or observed in any previous disordered model, Hermitian or non-Hermitian.

Referee 3 suggests that "the coexistence of ballistic transport and strong spectral localization" is a standard manifestation of non-reciprocity, asserting that this behavior comes from the fact that the initial state is simply driven toward the localization center of skin modes (boundaries in conventional NHSE, or bulk peaks for ENHSL). We believe this interpretation stems from the overly brief discussion in the original submission, which did not yet clarify the genuinely new dynamical mechanism at work here. In the revised manuscript, we have now substantially expanded and clarified this central point.

Our theoretical analysis shows that dynamics in the ENHSL regime arises from an interplay between intrinsic (Hermitian) ballistic propagation and disorder-induced amplification. However, this mechanism alone does *not* explain how ballistic transport can persist despite spectral localization. A naïve "excitation–funnelling" picture—briefly invoked by the referee and associated with Ref. [3]—would predict that the wave packet simply flows toward the global extremum of the random-walk landscape X_n, resulting in a final *dynamical localization* (attraction) around some erratic site in the bulk, not ballistic motion. The flaw in this intuitive "funneling" picture is crucial: although X_n indeed possesses a global maximum, its location can occur arbitrarily far from the initial position, and its distance fluctuates strongly between disorder realizations. Determining how far the wave packet propagates—and with what statistical law—is therefore highly nontrivial, and cannot be understood using the standard NHSE picture of localization at boundaries or interfaces. Instead, the correct description requires the *extreme-value statistics* of random walks, fundamentally different from the typical-fluctuation–based mechanisms governing conventional disordered NH dynamics. This leads to a qualitatively new physical regime: transport is controlled not by typical disorder but by *rare, sample-specific* excursions of a random walk. Equation (5) of our manuscript, derived analytically and confirmed experimentally, establishes that the dominant propagation site follows the universal Lévy–arcsine distribution,

and because of this law we can observe on average ballistic (rather than sub-ballistic or dynamical localization) transport. This finding uncovers an unexpected and previously unexplored connection between non-Hermitian transport at a topological transition and classical extreme-value statistics of random walks. To our knowledge, this connection is entirely new and has never been predicted or observed in any wave-transport setting.

To conclude, we believe that the combination of (i) the first experimental observation of a new form of localization (ENHSL) and (ii) the discovery of its unexpected ballistic transport regime in the globally reciprocal case, i.e. at a critical topological phase transition point, constitutes a genuinely impactful advance. These results shed new light on the fundamental relationship between eigenstate localization and transport in non-Hermitian disordered systems involving classical extreme-value statistics of random walks, and open a direction of broad relevance across classical and quantum wave platforms. For these reasons, we respectfully believe the work is suitable for publication in a high-impact journal such as Nature Communications.

Changes

In response to the referee's comments, we have made several substantial revisions to clarify the novelty and broaden the scope of the work. In particular, we have explicitly emphasized the distinction with respect to the previous PRL paper, highlighting that the present manuscript goes significantly beyond the original spectral theory by introducing and experimentally demonstrating a nontrivial dynamical regime whose explanation goes beyond the simple "funneling" picture of the NHSE, deeply involving extreme-value statistics and rare events of random walks. Specifically, we have clarified that the observation of ballistic transport and the emergence of Lévy–arcsine statistics in the globally reciprocal regime were neither predicted nor analyzed in the previous work, and represent a central, non-trivial new result of this study.

In addition, we have considerably extended the theoretical framework. A new section has been added at the beginning of the Methods, where we demonstrate that ENHSL is not restricted to the disordered Hatano–Nelson model considered in our experiments, but instead arises generically in a broad class of non-Hermitian systems obtained via imaginary gauge deformations of Hermitian parent lattices under the condition of global reciprocity. This generalization establishes the universality of ENHSL beyond specific model realizations and further strengthens the conceptual scope of the manuscript.

3.2 Comment 1

> The terminology "spectral localization" is confusing. I see clear spatial localization in the figures; but the spectra in Fig. 1 (schematic) and 3 (exp. and sim.) do not show anything special: they just form a continuum band on the complex energy plane. So what does

> “spectral” in this phrase imply?

Responses

We thank the Referee for raising this conceptual point. In the revised manuscript we now clarify the meaning of the term *spectral localization*, which in the theory of disordered systems has a precise and standard definition.

In the context of disordered Hamiltonians (for example, Anderson localization), it is important to distinguish between **spectral localization** and **dynamical localization**, which refer to different physical and mathematical properties:

Spectral localization refers to a property of the *spectrum* of the Hamiltonian. A disordered Hamiltonian is spectrally localized when all eigenstates are spatially localized, with a localization length determined by the inverse Lyapunov exponent. In our globally reciprocal model (i.e., for mean non-reciprocity $\overline{h} = 0$), all eigenvectors are spatially localized. However, the Lyapunov exponent vanishes, placing the system at a *critical* point in the usual classification of spectral localization. **Dynamical localization** refers instead to the time evolution of an initially localized excitation. A system is dynamically localized when such a wave packet remains localized in time and does not spread throughout the lattice.

In most *Hermitian* disordered systems, these two notions coincide: spectral localization implies dynamical localization, and vice versa. This equivalence follows from well-known sufficient conditions, in particular from the fact that the Lyapunov exponents of all eigenstates do not accumulate at zero. When the Lyapunov exponents *do* accumulate toward zero, one can instead observe transport (typically diffusive or subdiffusive), despite the presence of spatially localized eigenstates.

In *non-Hermitian* systems, by contrast, the relationship between spectral and dynamical localization is significantly more subtle. Our theoretical analysis, supported by the experiments, shows for the first time that a system can exhibit simultaneously:

- spectral localization of *all* eigenstates,
- ballistic transport *on average*,
- a spreading behavior governed by a Levy–arcsine law.

This coexistence is highly nontrivial. It cannot be understood from the standard non-Hermitian skin-effect picture: at $\overline{h} = 0$ the system is globally reciprocal, the NHSE is absent, eigenstates do not accumulate exponentially at boundaries or interfaces, and instead display more-than-algebraic localization at disorder-dependent positions. The emergence of ballistic spreading is instead rooted in the extreme-value statistics of symmetric random walks determined by the fluctuating sequence h_n.

We have now clarified in the manuscript that the adjective *spectral* in *spectral localization* refers to the nature of the Hamiltonian spectrum and the localization of its eigenstates. It does *not* imply that the plotted complex-energy spectrum must show an unusual visual pattern. As the Referee notes, the spectra in Figs. 1 and 3 form a continuum band in the complex plane, which is entirely compatible with spectral localization in the standard sense used in the mathematical theory of random operators.

Changes

Lines 47–51 in the revised manuscript. We explained that "spectral localization" means that all eigenstates of the Hamiltonian are spatially localized.

4 Comments from Reviewer 4

4.1 General comments

> The manuscript investigates a non-Hermitian chain with disordered nonreciprocal hopping. For comparison, the authors analyze the eigenstates and dynamical behavior relative to classical Anderson localization. They theoretically identify a distinct localization phenomenon—erratic non-Hermitian skin localization (ENHSL)—and corroborate it experimentally using an acoustic platform. However, despite the novelty of ENHSL, the underlying mechanism is not explained with sufficient clarity. The experimental measurement of Green's function matrix is also well developed in their previously published paper (Ref. 46) and does not, by itself, constitute a substantive experimental advance. For these reasons, I do not consider the work suitable for publication in Nature Communications, owing to limited experimental novelty and insufficient theoretical development. The following issues should be addressed before the manuscript is submitted to another journal.

Responses

We thank the referee for the careful reading of our manuscript and for raising these important concerns. We have taken the referee's comments very seriously, and in the revised version we have introduced substantial additions and clarifications that directly address the points raised. These revisions significantly expand both the theoretical framework and the physical interpretation of the phenomenon, and we believe they overcome the referee's criticisms and considerably strengthen the manuscript in terms of novelty, impact and broad relevance.

Below we summarize the key points.

First, the revised manuscript now contains a significantly strengthened and broadened theoretical analysis. In contrast to the original submission, we now provide a general and model-independent formulation of erratic non-Hermitian skin localization (ENHSL). In a new section added to the Methods, we show that ENHSL arises in a wide class of non-Hermitian lattices obtained by imaginary-gauge deformation of arbitrary Hermitian parent Hamiltonians, even in the presence of long-range hopping, multiple bands, and arbitrary unit cells, provided the global reciprocity condition is satisfied. This establishes, for the first time, the universality of ENHSL beyond the specific disordered Hatano–Nelson model considered in the experiments. These new theoretical developments go considerably beyond the scope of the earlier PRL work, and directly address the referee's concern regarding the underlying mechanism.

Second, the central result of the present work is not a repetition of the spectral analysis previously reported in Ref. 46, nor merely an experimental verification of the theory in the earlier PRL. Rather, the key discovery here is *a new dynamical regime* in the globally reciprocal limit, i.e. at the topological transition point where the conventional NHSE is absent. In this regime the

eigenstates are all spatially localized ("spectral localization"), but the wave packet nevertheless spreads *ballistically on average*, with the dominant propagation position obeying a Lévy–arcsine distribution. This remarkable coexistence of spectral localization and ballistic transport is entirely absent from the earlier PRL, was not predicted there, and cannot be understood using standard NHSE arguments. Instead, it reflects a fundamentally new transport mechanism governed by the extreme-value statistics and rare events in the theory of classical random walks. This conceptual advance is, in our view, one of the most important contributions of the work, and we have now emphasized and clarified it extensively in the revised manuscript.

Third, regarding the experimental methodology: while the Green's-function-based spectroscopic protocol used here builds upon our earlier work, the present experiment is not limited to spectral reconstruction. The same platform allows us to *simultaneously* measure both the full complex spectrum/eigenstates *and* the real-time wave-packet dynamics, enabling a unique one-to-one comparison between the static and dynamical signatures of ENHSL. This combined capability is essential for revealing the coexistence of irregular spectral localization with disorder-independent ballistic transport—a central result of the present work that cannot be accessed by spectral probes alone. Moreover, in the revised text we now provide a more balanced treatment of the literature on Green's-function spectroscopy and nonreciprocal acoustic couplings, as suggested by the referees.

Taken together, the major revisions substantially strengthen the manuscript: we now (i) provide a universal theoretical foundation for ENHSL, (ii) reveal and experimentally demonstrate a dynamical phenomenon not captured in previous theory, and (iii) clarify that the combined spectral and dynamical measurements go well beyond earlier uses of Green's-function-based techniques. For these reasons, we respectfully believe that the work presents a timely and conceptually significant contribution suitable for publication in a high-impact journal.

Changes

- Lines 43–51 in the revised manuscript. We revised the Introduction to illustrate the central physical question of the work. We now explicitly state that, although spectral localization is usually associated with dynamical localization or sub-ballistic transport, it remains an open and unconventional possibility whether ballistic transport can coexist with complete eigenmode localization.
- Lines 52–63 in the revised manuscript. We strengthened the conceptual positioning of ENHSL by clarifying that it occurs at a topological phase-transition point in globally reciprocal non-Hermitian lattices, and that it constitutes a universal disorder-induced localization mechanism beyond a specific Hatano–Nelson realization.
- Lines 64–86 in the revised manuscript. We revised the Introduction to emphasize the main experimental and conceptual advance of the present work: the first experimental

realization of ENHSL, together with the discovery that ensemble-averaged dynamics remain ballistic despite strong spectral localization.

- We expanded the "Model and conceptual overview" section to clarify the underlying mechanism of ENHSL. In particular, we now introduce the mean imaginary gauge field $\overline{h}$, explain its role in controlling global non-reciprocity and the conventional NHSE, and identify the globally reciprocal point $\overline{h} = 0$ as the transition point where ENHSL emerges.
- Lines 91–114 in the revised manuscript. We added a clearer distinction between the globally non-reciprocal and globally reciprocal cases. For $\overline{h} \neq 0$, the system exhibits the usual NHSE, including boundary-sensitive spectra, nonzero winding, exponential edge localization, and biased transport; for $\overline{h} = 0$, the random-walk landscape becomes unbiased and gives rise to subexponential, bulk ENHSL peaks governed by large excursions of X_n.
- We substantially expanded the main-text dynamics theory. We now show how the non-unitary gauge transformation maps the dynamics to a Hermitian ballistic propagator modulated by the random-walk envelope, and we explain why a simple "funnelling" picture toward the global maximum of X_n is insufficient.
- Lines 231–242 in the revised manuscript. We clarified that ENHSL transport is controlled by extreme-value statistics within the finite-time light cone, rather than by typical disorder fluctuations. This leads to the dominant-site selection rule and to the universal Lévy–arcsine distribution for $n_0(t)$, which we identify as a central theoretical prediction verified experimentally.
- We added a new Methods subsection, "General theoretical framework of the ENHSL", to provide a broader and more model-independent theoretical foundation. This section derives ENHSL from imaginary-gauge deformations of a general Hermitian parent lattice with arbitrary unit cell, long-range hopping, and multiple bands, thereby demonstrating that ENHSL is not specific to the stochastic Hatano-Nelson model.
- We further expanded the Methods derivation of ENHSL dynamics. The revised Methods now give the detailed gauge mapping, Bessel-function propagator, light-cone bound, normalized probability profile, and Lévy–arcsine derivation based on random-walk extreme-value statistics.
- Lines 67–85 in the revised manuscript. We revised the measurement-protocol section to clarify that the experimental advance is not Green's-function spectroscopy alone. Rather, the same acoustic platform now combines full complex-spectrum/eigenstate reconstruction with time-resolved wave-packet measurements for the same disorder realization, enabling a direct spectral–dynamical comparison that was not available from spectral measurements alone.

4.2 Comment 1

> What is the underlying mechanism of ENHSL? Specifically, what's the meaning of "the spectrum remains approximately real" (as noted in the caption of Fig. 1), and is the spectrum dependent on system size?

Responses

We thank the referee for this important question, which we have now clarified in the revised manuscript.

The underlying mechanism of ENHSL can be understood in terms of an imaginary-gauge deformation of a Hermitian parent lattice and the extreme value statistics of symmetric random walks. In the revised manuscript (see the new subsection in the Methods), we provide a general theoretical framework showing that a disordered non-Hermitian Hamiltonian of the form

$$\tilde{H}_{n,l} = H_{n,l} e^{h_n - h_l}$$

is related to the clean Hermitian Hamiltonian H by a non-unitary similarity transformation. The non-Hermitian Hamiltonian displays a topological phase transition as the average imaginary gauge field

$$\overline{h} = \lim_{N\to\infty} \frac{1}{N} \sum_{n=1}^{N} h_n$$

vanishes, as signaled by an abrupt change of a topological winding number (see e.g. Ref.[47] in our manuscript). When $\hat{h} \neq 0$, the system displays the usual NHSE, with the spectrum of $\tilde{H}$ strongly dependent on the boundary conditions (either OBC or PBC) and all eigenstates exponentially localized toward one lattice edge under OBC. Conversely, in the critical case $\hat{h} = 0$, corresponding to a globally-reciprocal system, the spectrum of $\tilde{H}$ – in the thermodynamic limit of lattice number site $N \to \infty$– is the same under PBC and OBC, it is entirely real, and the Hamiltonian eigenstates display the novel form of ENHSL: the eigenstates of the Hamiltonian are localized near the extreme values of the random walk $X_n = \sum_{l=0}^{n} h_l$, whose spatial location is strongly dependent on the specific disorder realization (i.e. 'erratic'). Localization is sub-exponential, which is a distinctive feature of ENHSL as compared to both NHSE and Anderson localization. This mechanism is now explicitly discussed in the revised manuscript.

The statement in the manuscript that "the spectrum remains approximately real" refers to the fact that, in the experiment, a small uniform imaginary shift is present due to intrinsic losses of the acoustic resonators and that the lattice site number N is not infinite. This results in a nearly real spectrum with a constant negative imaginary offset, rather than a strictly real one. We have clarified this point in the revised text.

Regarding the dependence on system size, in the thermodynamic limit $N \to \infty$ the spectra under

periodic and open boundary conditions coincide and are purely real under global reciprocity, as proven in the new Methods section. For finite systems, small deviations can arise due to finite-size effects and the specific disorder realization, but these corrections vanish as N increases. This explains the weak residual dependence on boundary conditions and system size observed in the experiments and simulations.

We hope that these additions clarify both the origin of the real spectrum and the physical mechanism underlying ENHSL.

Changes

- Lines 88–99 in the revised manuscript. We revised the “Model and conceptual overview” section to clarify the physical mechanism of ENHSL. We now introduce the mean imaginary gauge field $\overline{h}$, which quantifies the global non-reciprocity of the disordered Hatano-Nelson chain. We explicitly explain that $\overline{h} \neq 0$ gives the conventional NHSE, whereas the globally reciprocal condition $\overline{h} = 0$ is the transition point where the conventional NHSE disappears and ENHSL emerges.
- Lines 103–120 in the revised manuscript. We added a more detailed explanation of the ENHSL mechanism in terms of the random-walk landscape $X_n = \sum_\ell h_\ell$. In the globally reciprocal case, X_n is an unbiased symmetric random walk; therefore, the Lyapunov exponent associated with a macroscopic drift vanishes, preventing ordinary exponential edge accumulation. Instead, large excursions of X_n generate realization-dependent stochastic interfaces and subexponential bulk localization peaks, which is the defining mechanism of ENHSL.
- Figure 1 caption in the revised manuscript. We clarified the meaning of the statement that the spectrum remains “approximately real”.
- We expanded the Methods by adding a new subsection, “General theoretical framework of the ENHSL”, to justify the real-spectrum and boundary-insensitivity statements more generally. In the new Methods section, we also explicitly discuss the dependence on boundary condition and system size.

4.3 Comment 2

> In Fig. 1(c,e), the eigenstates display two dominant peaks localized at the left and right edges, and the dynamical responses show bidirectional wave-packet propagation, with the right peak being slightly larger. What accounts for this asymmetry? Were the results averaged over multiple disorder realizations?

Responses

We thank the referee for pointing this out. The apparent asymmetry in Fig. 1(c,e) is not a systematic effect, but arises from the fact that these panels are schematic illustrations of a *single representative disorder realization*, rather than ensemble-averaged results. In a globally reciprocal ENHSL lattice, the imaginary gauge field has zero mean and therefore does not impose a preferred direction. However, for any individual realization, the cumulative random-walk landscape $X_n = \sum_\ell h_\ell$ is generally asymmetric over the finite chain and can contain unequal positive and negative excursions. The dominant localization peaks and the transient wave-packet evolution therefore need not be left–right symmetric in a single realization. A mirror-related disorder realization would produce the opposite asymmetry, while the ensemble over many zero-mean disorder realizations is statistically unbiased.

We also clarify that the peaks sketched in Fig. 1(c) are intended to represent realization-dependent *bulk* ENHSL peaks, not conventional boundary skin modes. Their positions and relative amplitudes are sample-specific and are governed by the large excursions of the random-walk landscape. Similarly, the orange traces in Fig. 1(e) illustrate the time evolution for one representative realization, whereas the dashed curves $n = \pm\langle d(t)\rangle$ indicate the symmetric ensemble-averaged spreading envelope. Thus, the slight right-side dominance in the schematic is only an illustrative feature of the chosen realization and should not be interpreted as a net drift or as evidence of conventional NHSE.

To avoid this possible misunderstanding, we have revised the caption of Fig. 1 to explicitly state that panels (c) and (e) show a single representative realization and are not disorder-averaged. We have also clarified that the left-right asymmetry reflects realization-specific fluctuations of the zero-mean random-walk landscape, whereas ensemble averaging over many disorder configurations restores statistical left-right symmetry.

Changes

- We revised the caption of Fig. 1 to clarify that panels (c) and (e) are schematic illustrations of a single representative disorder realization, rather than disorder-averaged results.

4.4 Comment 3

> In Fig. 4, the spreading distance $d(t)$ for case (c) shows a markedly different trend from cases (a) and (b). Does this imply that ENHSL is highly sensitive to sample configuration?

Responses

We thank the referee for this important question. Yes, the single-realization dynamics of ENHSL is intrinsically sensitive to the specific disorder configuration, and this realization dependence

is precisely one of the defining features of the "erratic" localization regime. However, this sensitivity should not be interpreted as an instability or lack of robustness. Rather, it reflects the fact that the wave-packet dynamics is governed by the realization-specific random-walk landscape $X_n = \sum_\ell h_\ell$, especially by the dominant excursions of X_n within the finite-time light cone.

The different trend of $d(t)$ in Fig. 4(c) compared with Figs. 4(a,b) arises from the detailed structure of the corresponding random-walk landscape and eigenstate profile. In realization 3, the mode-summed intensity Ψ_n contains weak but non-negligible satellite structures away from the dominant peak, and the spatiotemporal evolution shows additional transient weight spreading to more distant sites. Since $d(t)$ is a second-moment-type observable, it is particularly sensitive to such weak weight at larger $|n|$. As a result, even a relatively small satellite component can produce a visibly different $d(t)$ curve, giving realization 3 a more extended and oscillatory spreading-distance profile than realizations 1 and 2.

We emphasize that this behavior is fully consistent with the ENHSL mechanism. In a single realization, the wave packet is drawn toward the dominant excursion of X_n, but if several comparable excursions or satellite structures lie within the light cone, the transient dynamics can involve multiple competing localization regions. This leads to sample-specific trajectories of $d(t)$, as seen in Fig. 4(a–c).

Most importantly, the main conclusion of the work does not rely on any single realization. The robust transport statement is made at the ensemble level: after averaging over many disorder realizations, the spreading remains ballistic, and the dominant-site statistics follow the Lévy–arcsine law. Thus, Fig. 4 illustrates the realization-dependent, erratic nature of ENHSL dynamics, whereas Fig. 5 establishes the statistically robust ensemble behavior.

Changes

- Lines 308–314 in the revised manuscript. We clarified in the "Experimental observation of single-realization dynamics" section that the spreading distance $d(t)$ can differ substantially between individual ENHSL realizations because it is sensitive to realization-specific satellite structures.

References

[1] Jahan Claes and Taylor L. Hughes. Skin effect and winding number in disordered non-Hermitian systems. *Phys. Rev. B*, 103(14):L140201, April 2021.

[2] Bikashkali Midya. Topological phase transition in fluctuating imaginary gauge fields. *Phys. Rev. A*, 109(6):L061502, June 2024.

[3] Sebastian Weidemann, Mark Kremer, Tobias Helbig, Tobias Hofmann, Alexander Stegmaier, Martin Greiter, Ronny Thomale, and Alexander Szameit. Topological funneling of light. *Science*, 368(6488):311–314, April 2020.

Response to Reviewer 3 (second round)

We thank the Reviewer for the additional comments and for carefully reconsidering our revised manuscript. While we appreciate the Reviewer’s perspective, we respectfully maintain that the present work provides a substantial advance beyond a straightforward experimental verification of the recently proposed ENHSL mechanism.

First, irrespective of the transport aspect, the manuscript reports the first experimental observation of erratic non-Hermitian skin localization, a fundamentally different localization mechanism that is distinct from both Anderson localization and the conventional non-Hermitian skin effect. We believe that establishing the experimental existence of such a previously unobserved localization regime already represents a noteworthy result.

Second, we respectfully disagree that the observed ensemble ballistic transport can be understood within the simplified "funneling" picture suggested by the Reviewer. Such an intuitive picture may explain why, in an individual realization, the wave packet is attracted toward disorder-selected amplification maxima, but it does not explain why the ensemble-averaged transport remains ballistic rather than becoming diffusive or sub-ballistic, as one might naturally expect for transport in a random landscape. As clarified in the revised manuscript, this behavior instead originates from a fundamentally different mechanism governed by the extreme-value statistics of symmetric random walks within the finite-time light cone. The resulting Lévy-arcsine statistics provide a quantitative and predictive theoretical framework that, to our knowledge, has not previously been recognized in the context of wave transport. We believe that this theoretical insight is essential for understanding the observed ballistic scaling and cannot be inferred either from the conventional NHSE picture, which relies on a net nonreciprocal bias, or from a qualitative "funneling" argument alone in the globally reciprocal regime considered here.

These points are already clarified in the revised manuscript, where we have taken care to present our conclusions in a balanced manner without overstating our claims. We respectfully maintain that the combination of the first experimental realization of ENHSL together with the identification and experimental verification of its unexpected transport mechanism constitutes a significant conceptual and experimental advance.

Yun Jing
On behalf of all authors
Junly 26, 2026
